# Predicting HIV-1 transmission and antibody neutralization efficacy *in vivo* from stoichiometric parameters

**DOI:** 10.1371/journal.ppat.1006313

**Published:** 2017-05-04

**Authors:** Oliver F. Brandenberg, Carsten Magnus, Peter Rusert, Huldrych F. Günthard, Roland R. Regoes, Alexandra Trkola

**Affiliations:** 1 Institute of Medical Virology, University of Zürich, Zurich, Switzerland; 2 Division of Infectious Diseases and Hospital Epidemiology, University Hospital Zurich, Zurich, Switzerland; 3 Institute of Integrative Biology, ETH Zurich, Zurich, Switzerland; University of Wisconsin-Madison, UNITED STATES

## Abstract

The potential of broadly neutralizing antibodies targeting the HIV-1 envelope trimer to prevent HIV-1 transmission has opened new avenues for therapies and vaccines. However, their implementation remains challenging and would profit from a deepened mechanistic understanding of HIV-antibody interactions and the mucosal transmission process. In this study we experimentally determined stoichiometric parameters of the HIV-1 trimer-antibody interaction, confirming that binding of one antibody is sufficient for trimer neutralization. This defines numerical requirements for HIV-1 virion neutralization and thereby enables mathematical modelling of *in vitro* and *in vivo* antibody neutralization efficacy. The model we developed accurately predicts antibody efficacy in animal passive immunization studies and provides estimates for protective mucosal antibody concentrations. Furthermore, we derive estimates of the probability for a single virion to start host infection and the risks of male-to-female HIV-1 transmission per sexual intercourse. Our work thereby delivers comprehensive quantitative insights into both the molecular principles governing HIV-antibody interactions and the initial steps of mucosal HIV-1 transmission. These insights, alongside the underlying, adaptable modelling framework presented here, will be valuable for supporting *in silico* pre-trial planning and post-hoc evaluation of HIV-1 vaccination or antibody treatment trials.

## Author summary

Successful solicitation of the potential of neutralizing antibodies for HIV-1 prevention will require a deepened understanding of HIV-1 transmission and antibody neutralization. In this study, we experimentally determined molecular parameters of the HIV-1-antibody interaction, and subsequently used this knowledge to devise a mathematical model of HIV-1 infection and antibody neutralization *in vivo*. First, we experimentally confirmed that binding of one antibody per HIV-1 envelope trimer is sufficient for trimer neutralization. This finding, in combination with the number of trimers per HIV-1 virion, the number of trimers required for virus entry, and the affinity of antibody-trimer binding, enabled precise modelling of HIV-1 antibody neutralization. We employed our model for a post-hoc analysis of non-human primate infection studies, thereby obtaining estimates of HIV-1 neutralization *in vivo* and the probability for a single HIV-1 virion to initiate host infection. We further modelled HIV-1 infection and antibody neutralization during male-to-female transmission in the human host, which delivered estimates for the likelihood of HIV-1 transmission per sexual act and predictions of protective mucosal antibody concentrations. The quantitative insights into HIV-1 infection and antibody neutralization derived here, spanning from the molecular to the systemic level, contribute to a refined understanding of HIV-1 transmission and may prove useful for pre-study planning or post-hoc analyses of HIV-1 clinical trials and vaccine studies.

## Introduction

Recent years have seen tremendous success in the isolation and characterization of broadly neutralizing antibodies (bnAbs) from selected HIV-1 infected patients. By binding to the HIV-1 envelope glycoprotein trimer (Env), bnAbs succeed to neutralize a majority of circulating HIV-1 strains. It is assumed that the elicitation of antibodies will constitute a crucial component of a successful HIV-1 vaccination strategy, and known bnAbs are intensely explored as templates for HIV-1 vaccine development [1–5]. Indeed, it has been conclusively demonstrated in animal models that passive immunization with bnAbs can protect against virus challenge, delay viral rebound and transiently lower viremia [6–19]. Furthermore, passive immunization in human patients demonstrated an impact of bnAbs on established HIV-1 infection [20–22], underscoring the potential relevance of bnAbs to prevent or treat HIV-1 infection.

**Fig 1:**
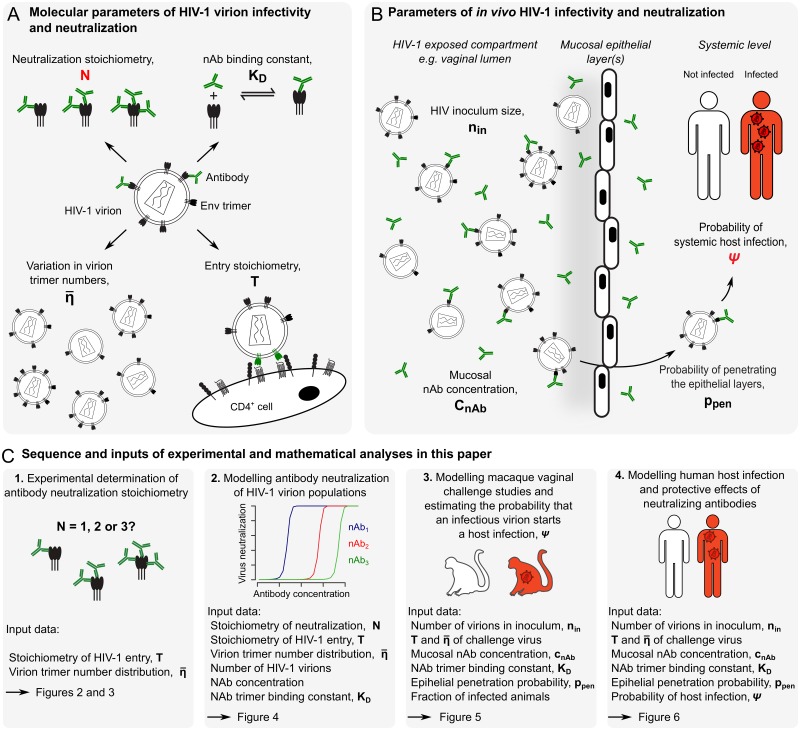
Parameters governing the interaction between HIV-1 and neutralizing antibodies and study layout. (A) Molecular parameters included in this study to define HIV-1 infectivity and nAb neutralization. (B) Parameters included in this study to define *in vivo* HIV-1 infection and nAb neutralization. All parameters used for modelling are highlighted in bold and are summarized in S1 Table. Parameters estimated in this study, notably the stoichiometry of trimer neutralization, N, and the probability of an infectious virion to start a host infection, ψ, are highlighted in red. (C) Shown here is the sequence of experimental and mathematical analyses in this study, starting with the experimental estimation of N and extending to the modelling of human mucosal HIV-1 transmission.

However, despite this wealth of information on the protective effects of bnAbs *in vivo*, key parameters of the HIV-1 nAb interaction and HIV-1 host-to-host transmission remain ill-defined. This concerns both fundamental molecular aspects of Env trimer-nAb binding and systemic factors of mucosal HIV-1 transmission. Importantly, comprehensive knowledge of the molecular and systemic parameters governing HIV-1 transmission and nAb neutralization would empower *in silico* modelling of nAb activity and be instrumental to guide vaccine development or nAb treatment trials [23, 24]. We thus propose that precise numerical quantification of the parameters that steer nAb efficacy and *in vivo* HIV-1 transmission is needed. Moving towards this aim, we report here on a combined experimental-mathematical analysis providing comprehensive quantitative insight into mucosal HIV-1 transmission and nAb neutralization (Fig 1).

Starting at the molecular level, the first question we addressed regards the number of nAbs required to neutralize each HIV-1 Env trimer (the *stoichiometry of neutralization*, N). This number, in combination with the mean number of trimers per HIV-1 virion ($\bar{\eta }$) and the number of trimers required for virus entry (T, Fig 1A) defines the number of nAbs needed to neutralize single HIV-1 virions or entire virion populations [25–31]. While such stoichiometric definitions may appear academic given the well-known potential of nAbs to neutralize HIV-1, we show here that these parameters are indeed crucial for an in-depth understanding of HIV-1 nAb neutralization and enable mathematical predictions of nAb neutralization potency.

Moving to the systemic level, we next addressed uncertainties in our understanding of mucosal HIV-1 transmission. Non-human primate studies revealed the interplay between nAb potency, *in vivo* nAb concentrations, and the resulting susceptibility or protection against virus infection [6–19]. However, a detailed systemic understanding of the mucosal infection process and the factors resulting in nAb protection from infection, ideally down to the single-virion level, are missing. Utilizing our stoichiometric model framework we performed a post-hoc analysis of selected animal studies, and obtained precise quantitative insight into mucosal nAb neutralization and the probability for single infectious HIV-1 virions to establish a systemic host infection.

Lastly, significant uncertainty is associated with the process and the probabilities of mucosal HIV-1 transmission in the human host via sexual contact. Per-exposure risk estimates of HIV-1 transmission vary widely, and uncertainty prevails regarding the concentration of nAbs in genital mucosal tissues that would provide protection from infection. This is not surprising, given the difficulties in estimating these parameters directly in the human population [32, 33]. Thus, in a final step we build on all previously determined parameters to model human HIV-1 penile-vaginal transmission. This analysis yielded predictions of HIV-1 male-to–female per intercourse transmission probabilities that match empirical data, and provided estimates of mucosal nAb concentrations expected to provide protection from HIV-1 infection.

## Results

We devised an experimental and mathematical framework to analyze interactions between HIV-1 virions and nAbs both on a molecular level and during HIV-1 transmission *in vivo*. Specifically, we (i) investigated the stoichiometric parameters of nAb binding to Env trimers on virions leading to neutralization, (ii) modelled nAb neutralization and HIV-1 infectivity both *in vitro* and in macaque passive nAb immunization virus challenge studies, and (iii) modelled HIV-1 transmission risk and protective effects of nAbs in the human host during penile-vaginal sexual contact. Our model framework relies on a defined set of parameters, retrieved from the literature or determined in this study (Fig 1A and 1B and S1 Table). Within our analytical framework, we conceptualize HIV-nAb interactions as follows:

(i) To model whether an HIV-1 virion to which a certain number of nAbs are bound is still infectious we expand previously developed stoichiometric models [26, 29, 31, 34, 35]. These models rely on the distribution of Env trimer numbers among HIV-1 virions (*η*), the number of trimers required for virus entry into a target cell (the entry stoichiometry, T) and the number of Env trimer subunits that nAbs must bind to neutralize the trimer (the neutralization stoichiometry, N). With these parameters we determine the average number of nAbs required for HIV-1 virion neutralization.
(ii) To transfer the *number* of nAbs required for virion neutralization into the more meaningful parameter of nAb *concentration*, we utilize nAb trimer binding affinity, K_D_. We model the nAb-Env interaction as a binding reaction depending on nAb trimer binding constant (K_D_) and nAb concentration (c_Ab_).
(iii) Modelling the impact of nAbs in preventing mucosal HIV-1 transmission requires knowledge of nAb concentrations at the port of entry (c_Ab_ in vaginal secretions and tissue), the HIV-1 inoculum size (n_in_), the probability of virions to penetrate the epithelial layers (p_pen_) and the probability that one infectious virion successfully initiates host infection (ψ).

In Fig 1C, we depict the sequence of experimental and mathematical approaches in this study, highlighting the parameters required for each successive modelling step.

### Estimating the stoichiometry of HIV-1 trimer neutralization, N

The topic of HIV-1 neutralization stoichiometry has previously been studied experimentally and computationally, with N = 1 being the most common conclusion [26, 29, 34]. However, ambiguity in these estimates prevailed, predominantly due to previously poorly defined parameters of HIV-1 entry stoichiometry, T, and mean virion trimer number, $\bar{\eta }$, which are essential for the analysis of N [27–29]. Here, we utilized recently determined values of T and $\bar{\eta }$ [28] to conclusively estimate N across different HIV-1 strains and bnAbs identified in recent years, for which estimates of N are currently lacking. As precise information on N across nAbs is indispensable for an in-depth numerical understanding of HIV-1 nAb neutralization (Fig 1A), we set out to estimate N for a range of nAbs and HIV-1 strains.

**Fig 2:**
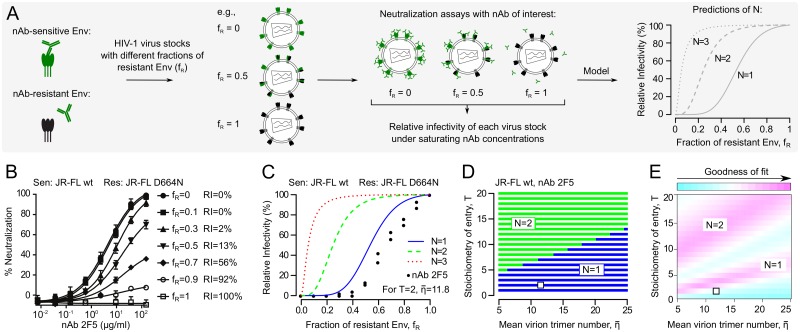
Estimating the stoichiometry of HIV-1 nAb neutralization (N) with mixed trimer assays. (A) Scheme depicting the combined experimental-mathematical approach employed here to estimate N. (B) Neutralization by nAb 2F5 of mixed trimer HIV-1 pseudovirus stocks containing the indicated ratios of JR-FL wt (2F5 sensitive) and JR-FL D664N (2F5 resistant) Envs. The relative infectivity (RI) of each stock is given by the percentage of target cell infection (i.e., the inverse of % neutralization) under saturating 2F5 nAb concentrations. (C) Theoretical model predictions of the relation between N (N = 1, 2 or 3; colored lines) and RI of virus stocks with different fractions of neutralization-sensitive to resistant Env (f_R_). The experimental JR-FL nAb 2F5 RI data from (B) are plotted as black dots, representing two independent experiments. Data fitting resulted in an estimate of N = 1, assuming T = 2 and $\bar{\eta }=11.8$ trimers for each JR-FL virion (S3 Table). (D) Robustness analysis for the N = 1 estimate of HIV-1 strain JR-FL and nAb 2F5 against variation in T and $\bar{\eta }$. Blue areas show combinations of T and $\bar{\eta }$ resulting in estimates of N = 1, green areas show combinations of T and $\bar{\eta }$ that would result in estimates of N = 2. The actual values of T and $\bar{\eta }$ for JR-FL (see above) are marked by the white dot, indicating that the N = 1 estimate is robust. (E) Since the model fit shown in (C) for JR-FL and nAb 2F5 is imperfect, we analyzed the goodness-of-fit. A slightly reduced $\bar{\eta }$ or slightly increased T compared to those used for the analysis (white dot, S3 Table) could improve the model fit to the experimental data.

To estimate N, we measured nAb neutralization of HIV-1 pseudoviruses carrying mixed trimers of nAb-sensitive and resistant Envs and analyzed the data with mathematical models (Fig 2A). Relative virus infectivities (RI) under saturating nAb concentrations were set in relation to the fraction of resistant Env (f_R_), shown for nAb 2F5 with Envs JR-FL wt (2F5 sensitive) and mutant JR-FL D664N (2F5 resistant) (Fig 2B, S1 Fig, S2 Table). Taking the JR-FL entry stoichiometry (T = 2) and mean trimer number per virion ($\bar{\eta }=11.8$) into account (S3 Table), our model predicts different RI profiles for different N (Fig 2C). Mathematical analysis of the experimental data indicated a neutralization stoichiometry of N = 1 for JR-FL by nAb 2F5 (Fig 2C). We confirmed the robustness of the N = 1 estimate against variation in virus entry stoichiometry (T) and mean virion trimer number ($\bar{\eta }$) by sensitivity analyses (Fig 2D). To investigate the observed deviations of the model fit from the experimental data (Fig 2C), we performed a goodness-of-fit analysis (Fig 2E). This analysis indicated that lower values of T and/or $\bar{\eta }$ could result in better curve fits. While we expect T to be constant for each viral strain, fluctuations in $\bar{\eta }$ from experiment to experiment are conceivable and provide a potential explanation for the deviation between experimental data and model predictions. In addition, mean virion trimer numbers of a given virus preparation may decrease over time as spontaneous Env shedding can occur resulting in non-functional trimers [36].

To further test variation in T and $\bar{\eta }$ on predictions of N, we assessed N for nAb 2F5 against Env variants of HIV-1 strains JR-FL and NL4-3 that differ in T or $\bar{\eta }$ (S2A–S2F Fig, S3 Table) [28, 37]. We indeed observed divergent RI profiles upon variation in T or $\bar{\eta }$ as predicted by our model. However, for all Env variants tested we retrieved estimates of N = 1.

To explore if nAb avidity influences estimation of N, we compared nAb 2F5 IgG and Fab fragment. Both yielded overlapping RI profiles for strains JR-FL and NL4-3 and identical estimates of N = 1 (S2G–S2I Fig), confirming that estimation of N is independent of nAb valency.

### NAb binding to a single Env subunit is sufficient for trimer neutralization

**Fig 3:**
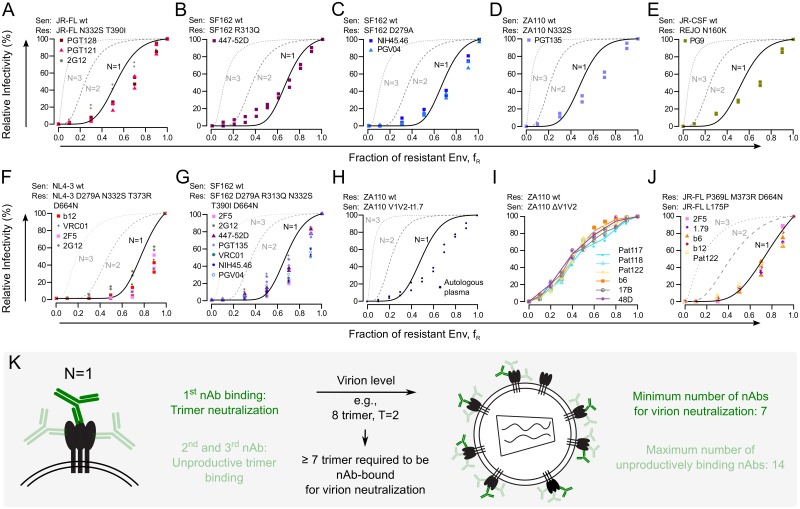
N = 1 is a general feature of broadly neutralizing antibodies and polyclonal HIV-1^+^ patient plasma IgG. (A) to (E) Mixed trimer neutralization setups with the Envs and nAbs indicated in each panel, yielding estimates of N = 1 in all cases. (F) to (G) Envs with multiple nAb resistance mutations allowed parallel assessment of various nAbs on the same set of mixed trimer virus stocks. Mathematical analysis indicated N = 1 in all cases. (H) Mixed trimer setup with Envs ZA110 wt and ZA110-V1V2^1.7^, the latter being sensitive to autologous HIV-1^+^ patient plasma. The model fit to the experimental data (black dots) yielded a plasma neutralization stoichiometry of N = 1. (I) Mixed trimer setup with Envs ZA110 wt and ZA110 ΔV1V2 and nAbs b6, 17B and 48D and three heterologous HIV-1^+^ patient plasma (Pat117, Pat118 and Pat122). The Envs in this setup are not infectivity-matched precluding mathematical analysis, but graphic comparison indicates equal N of nAbs and patient plasma. (J) Mixed trimer setup with Envs JR-FL L175P and JR-FL P369L M373R D664N and nAbs b6, 1.79, 2F5, b12 and heterologous patient plasma SP122, yielding equal estimates of N = 1. All data points depict two independent experiments. (K) N = 1 is a universal feature of all anti-HIV nAbs tested here. This defines decisive numerical requirements of HIV-1 virion neutralization by antibodies.

Having validated our approach to estimate N (Fig 2), we sought to obtain a comprehensive analysis of N for various HIV-1 nAbs including VRC01, NIH45-46, PGV04, b12, PGT121, PGT128, PGT135, 2G12, PG9, PGT145, and 2F5 (S4 Table). We therefore generated a panel of Env mutants in five divergent HIV-1 strains with single or combined nAb resistance mutations (S1 Fig). Several of these Env mutants showed a significant reduction in virus infectivity (S2 Table). This is critical to note, as matched virus entry parameters, notably T and $\bar{\eta }$, are a prerequisite for the analysis of N (S2 Fig). Indeed, we observed that strong infectivity differences between nAb-resistant and sensitive Envs in mixed trimer assays result in substantial deviations of the RI profiles that prohibit determination of N (S3 Fig). We thus restricted our analysis to mixed trimer assays with nAb sensitive-resistant Env pairs of comparable infectivity (≤ 2-fold infectivity difference). In a direct comparison of eight different nAbs across five Env mixed trimer combinations, we thereby obtained a consistent estimate of N = 1 irrespective of nAb epitope specificity, potency or HIV-1 strain (Fig 3A–3E, S4 Fig). The estimate of N = 1 was confirmed by bootstrap analyses (S5 Fig) and goodness-of-fit plots across all analyzed Env-nAb pairings (S6 Fig). As shown before (Fig 2E), the goodness-of-fit would in many cases be improved for lower values of T and/or $\bar{\eta }$, possibly indicating slight deviations in these parameters within the experimental setup.

In the above analysis we employed a hard threshold model in which, according to our analyses, binding of one nAb to a trimer results in complete loss of function. Soft threshold models, which allow for partial loss of trimer functionality upon nAb binding, were introduced in earlier studies by us and others [26, 29]. To investigate how a soft threshold model would fit to our current data set, we re-analyzed our data accordingly [26]. Here, we allow the probability of a virion to infect a cell to scale with the number of functional trimers (a soft threshold for virus entry) and the functionality of a trimer to decrease by successive nAb binding (a soft threshold for neutralization). Our analysis revealed that the functionality loss of a trimer upon nAb binding to one subunit is dominant, that is, a soft threshold model is not supported (S7 Fig). This finding thereby underscores the assumption of a hard threshold for the stoichiometry of HIV-1 nAb neutralization.

To validate that N = 1 across all nAbs tested here, we tested a set of nAbs targeting different epitopes against Env variants with multiple nAb escape mutations, allowing parallel analysis of diverse nAbs on the same set of mixed trimer virus stocks (Fig 3F and 3G, S8 Fig, S2 Table). Two Env combinations (Fig 3F and 3G) were infectivity matched and allowed mathematical analysis of N, yielding N = 1 for all nAbs tested. To derive an estimate of N in the non-infectivity matched setups we included nAb 2F5 throughout, as we previously established that it neutralizes with N = 1 (Fig 2). Furthermore, nAb PG9, which is known to bind with only one nAb per trimer [38, 39], was included in two of these setups (S8 Fig). As 2F5 and PG9 yielded highly similar curves compared to the other nAbs, we conclude that our analysis univocally inferred N = 1 for all probed nAbs.

Regarding this consistent N = 1 estimate across all nAbs tested, we asked whether non-antibody Env inhibitors would show a similar neutralization behaviour. To test this, we generated Env mutants resistant against the peptide T-20, a clinically used HIV-1 entry inhibitor targeting the Env gp41 subunit [40]. Using mixed trimer assays, we found that T-20 neutralizes with an N = 1 stoichiometry (S9 Fig). This indicates that, regardless of inhibitor type, interference with one Env trimer subunit is sufficient for HIV-1 trimer neutralization.

### Antibodies in HIV-1^+^ patient plasma neutralize with a N = 1 stoichiometry

Antibody responses in the majority of HIV-1 infections are largely ineffective in neutralizing the virus within the patient, and bear only limited neutralization potency and breadth against other HIV-1 strains. In contrast, the bnAbs available to date were isolated from rare HIV-1^+^ patients with high HIV-1 neutralization potency and breadth [41–43]. In our stoichiometry analysis, these bnAbs uniformly showed N = 1 (Fig 3A–3G). This raises the question if weakly neutralizing Abs as most commonly elicited during HIV-1 infection require a higher N, and whether N = 1 is a distinguishing feature of bnAbs. To test this, we determined N of the polyclonal antibody mix in HIV-1^+^ patient plasma. We first tested plasma from an individual with typical HIV-1 neutralization escape [44] and corresponding plasma neutralization-resistant and sensitive Env variants (ZA110 wt and ZA110-V1V2^1.7^, respectively; S2 Table). Mixed trimer assays yielded N = 1 for the plasma Abs of this individual (Fig 3H). We further tested plasma from three chronically HIV-1 infected individuals (Pat117, Pat118, Pat122) showing only weak HIV-1 neutralization activity. We tested these plasma on mixed trimer stocks of Envs ZA110 wt (resistant) and ZA110 ΔV1V2 (sensitive) and further included weakly neutralizing Abs b6, 17b and 48d in this analysis (Fig 3I). Comparison of the RI profiles indicated identical N for all probed plasma and nAbs. Additionally, plasma Pat122 neutralized HIV-1 strain JR-FL in an infectivity-matched setup with N = 1, as did several nAbs (Fig 3J).

Thus, irrespective of nAb potency, breadth or epitope specificity, neutralization of HIV-1 trimers requires only a single Env subunit to be bound by antibody (Fig 3K). This estimation of N defines numerical requirements for HIV-1 antibody neutralization [27, 29–31, 45], which we employed in subsequent modelling steps to assess HIV-1 virion population neutralization *in vitro* and *in vivo*.

### *In silico* prediction of nAb inhibitory capacity

**Fig 4:**
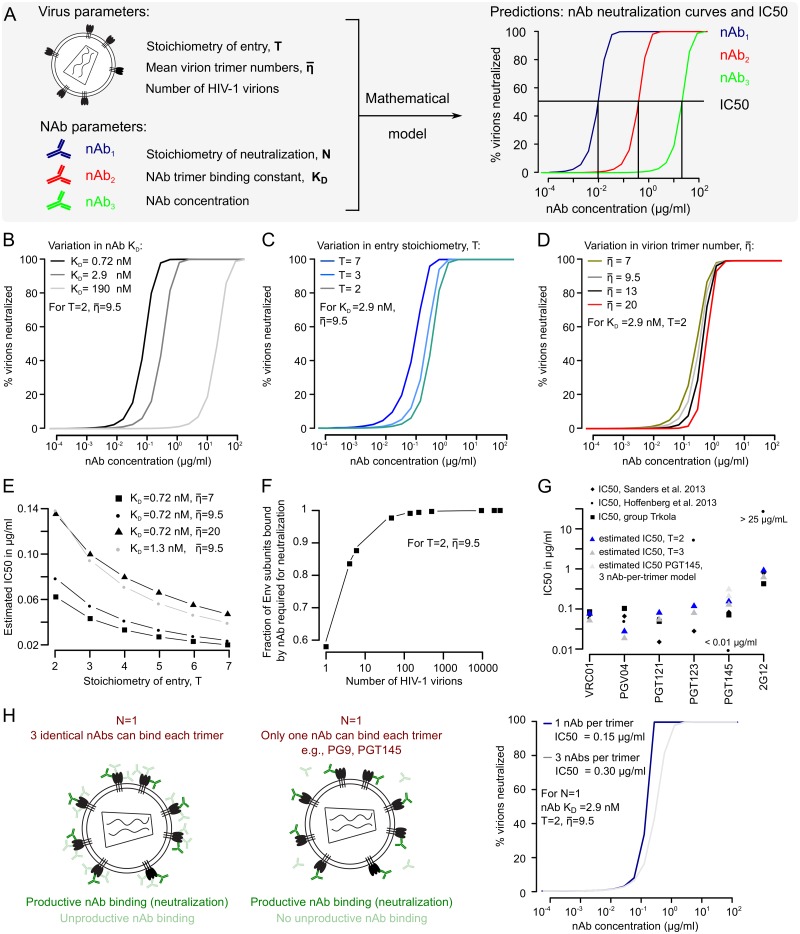
Predicting HIV-1 antibody neutralization curves and IC50. (A) Scheme depicting the virus and nAb-specific parameters necessary to predict HIV-1 neutralization by nAbs *in silico*. (B) to (D) Predicted HIV-1 nAb neutralization curves in dependence of (B) nAb K_D_, (C) HIV-1 entry stoichiometry, T, and (D) mean viron trimer number, $\bar{\eta }$. (E) Dependence of nAb IC50 on the number of Env trimers required for HIV-1 cell entry, T. (F) Relation between HIV-1 virion population size and the fraction of Env subunits that need to be bound by nAb to achieve virus population neutralization. (G) Comparison of experimental nAb IC50 values measured with HIV-1 strain BG505 [47, 48] (S5 Table) and IC50’s predicted by our model using BG505-specific T, $\bar{\eta }$ and K_D_ [49] values (S3 and S5 Tables). For nAb PGT145 we assumed that only 1 nAb can bind per trimer; the PGT145 IC50 estimates with the 3-nAb-per-trimer model, used for all other nAbs, are shown in light grey (see also panel H). (H) For most nAbs, we assume that three nAbs can bind each trimer, while binding of one nAb is already sufficient for neutralization (N = 1). Binding of nAbs to a trimer that is already neutralized constitutes unproductive binding and may reduce antibody concentrations to a subcritical level [27]. To quantify this effect, we predicted neutralization by two nAbs with equal K_D_, but assuming that either three nAbs can bind each trimer, or that nAb binding is anti-cooperative as observed for PG9/PGT145-like nAbs, which are known to occupy only one of three potential epitopes per trimer. We observed a slight neutralization advantage for PG9/PGT145-like nAb binding behavior, manifested as an approximately two-fold reduction in predicted nAb IC50.

We previously established a mathematical framework that predicts the number of nAbs required to neutralize a given HIV-1 virion population based on the stoichiometry parameters N, T and $\bar{\eta }$ [27]. The conclusive estimation of N (Figs 2 and 3), together with previously determined parameters T and $\bar{\eta }$ [28], now enabled us to use our framework for quantitative predictions of nAb neutralization. We extended the framework by including the affinity of nAb binding to Env trimers (represented by the binding constant, K_D_) [35, 46] to predict the fraction of neutralized HIV-1 virions for given nAb concentrations. In essence, this allowed us to simulate HIV-1 nAb neutralization curves *in silico* (Fig 4A).

For this analysis we utilized nAb binding constants, K_D_, recently reported for the HIV-1 strain BG505 SOSIP trimer [49]. We employed these K_D_ values together with T and $\bar{\eta }$ of BG505 (S3 Table) and N = 1 to model nAb neutralization of an HIV-1 BG505 virion population (Fig 4B–4D). As expected, we found that nAbs with high Env binding affinity (low K_D_) are predicted to require lower concentrations to achieve virion population neutralization (Fig 4B). The required nAb concentrations increase slightly with fewer trimers needed for HIV-1 entry (low T) (Fig 4C) and higher virion trimer content (high $\bar{\eta }$) (Fig 4D). The latter two trends can be rationalized as follows: in both cases (a small T or a high $\bar{\eta }$), more nAbs will be needed to neutralize a given virion population, resulting in higher predicted nAb concentrations.

The relation between nAb trimer binding and HIV-1 virion population neutralization is influenced by all parameters included in our model (Fig 4A). Especially the influence of T and $\bar{\eta }$ on nAb neutralization predictions should not be underestimated. This is highlighted in Fig 4E, depicting estimated values of nAb concentrations required for 50% virus population neutralization (IC50) in dependence on T and $\bar{\eta }$.

In addition to predicting nAb neutralization curves and IC50 values, we determined nAb concentrations required to achieve sterilizing neutralization of HIV-1 virion populations. Due to unproductive nAb binding to Env trimers (Fig 4H) [27], the fraction of Env subunits required to be bound by nAb for sterilizing neutralization of a virion population increases with virion population size (Fig 4F). Likewise, the predicted nAb concentrations required for sterilizing neutralization of virion populations increase with virion population size (S10A–S10C Fig). As shown in Fig 4B–4D for nAb neutralization curves, these nAb concentrations are influenced by nAb K_D_, T and $\bar{\eta }$ (S10A–S10C Fig). Of note, only nAb K_D_ has a direct linear relationship with sterilizing nAb concentrations, while the influence of T and $\bar{\eta }$ follows non-linear relations (S10D–S10F Fig).

We noted that our predicted neutralization curves show steeper slopes than commonly observed in HIV-1 neutralization assays (S11A Fig). We thus asked which parameters of our model could explain this deviation, and found that assuming broader virion trimer number distributions (i.e., higher variance in trimer numbers between virions) results in less steep predicted neutralization curves (S12 Fig). Of note, we also observed that our predicted curves are in closer agreement to *in vitro* neutralization data obtained with replication-competent virus and PBMC target cells (S11B Fig). While a detailed analysis of this relation and the underlying parameters is beyond the scope of this manuscript, this observation may be taken as indication that our model predicts neutralization curves better for replication-competent virus than for pseudovirus preparations.

To test the predictive power of our *in silico* approach, we compared experimentally derived IC50 values of HIV-1 strain BG505 and nAbs VRC01, PGV04, PGT121, PGT123, PGT145 and 2G12, originating from *in vitro* experiments by three independent laboratories [47–49], with nAb IC50 values estimated by our model (Fig 4G, S11 Fig, S5 Table). Our predicted IC50 values for nAbs VRC01 and PGT121 were in close agreement with the measured values. For nAbs PGT123 and 2G12, we observed wide variations in experimentally derived IC50 values; interestingly, the IC50 values predicted by our model lay in between these values. For nAbs PGV04 and PGT145, our estimated IC50s are slightly lower and higher than experimentally determined IC50s, respectively, though not far off (see below for a detailed discussion of the PGT145 IC50 estimation). This good agreement between experimental and predicted nAb IC50s highlights that the parameters included in our model (N, T, $\bar{\eta }$, nAb K_D_ and nAb concentration) capture relevant steps of HIV-1 virion neutralization. Of note, our model performs significantly better than a simpler model based on nAb K_D_ alone (S13 Fig).

We also derived predictions of nAb neutralization for scenarios of N = 1 but assuming that exclusively one nAb can bind one of the three epitopes displayed on each trimer (a binding behavior described for nAbs PG9, PG16 or PGT145 [38, 39, 49]). We compared these predictions to our standard model where we assume that all three trimer subunits can potentially be bound, although only one Env subunit needs to be bound to achieve neutralization. In this case, binding of the second and third nAb represents unproductive binding of nAbs, since the trimer is already neutralized by binding of the first nAb (Fig 3K). Importantly, we assume that also for PG9-like nAbs, initially the same number of epitopes is present as for “typical” nAbs (i.e., three per trimer), with the difference that binding of PG9-like nAbs shows negative cooperativity: after binding of the first antibody the remaining two epitopes are inaccessible. In our analysis, we assume two nAbs with the same K_D_ for their protomeric epitopes, but either a three-nAb-per-trimer or one-nAb-per-trimer binding behavior (Fig 4H). According to our predictions, binding of only one nAb per trimer results in a 2-fold decreased IC50, thereby demonstrating the effect of unproductive nAb binding (Fig 4H). Intriguingly, the PGT145 IC50 predictions using this model are closer to experimental PGT145 IC50 values than the predictions obtained with the standard model (Fig 4G). This indicates that nAbs with a one-nAb-per-trimer binding behavior should have a slight neutralization advantage, especially under conditions of low nAb concentrations.

### Estimating the *in vivo* efficacy of passive nAb immunization and single-virion infectivity

**Fig 5:**
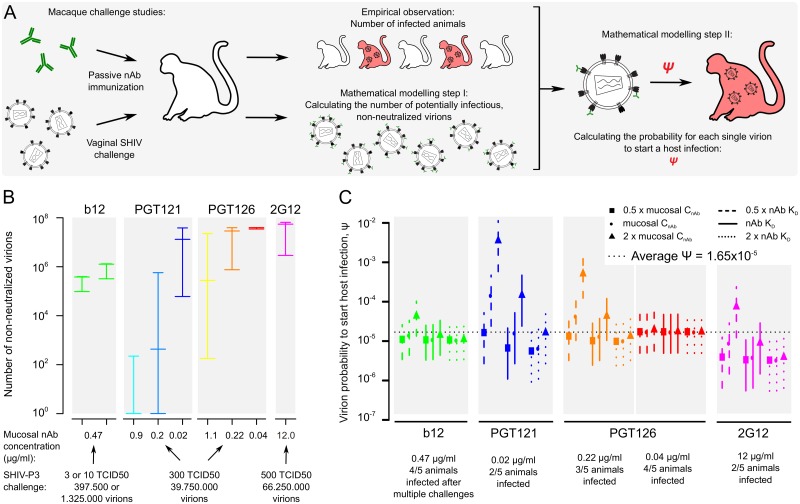
Antibody neutralization and host infection in macaque passive immunization challenge studies. (A) Scheme depicting our approach of post-hoc analysis of macaque passive nAb immunization vaginal virus challenge studies. The approach delivers both estimates of *in vivo* virus inoculum neutralization and the probability of each infectious virion to start a host infection. (B) Total number of SHIV-P3 virions remaining infectious in the four analysed studies, obtained by multiplying the study-specific SHIV-P3 virus inoculum sizes with the fraction of virions remaining non-neutralized by nAbs. Bars depict the lowest, mean and highest number of potentially infectious virions, based on different assumptions of nAb K_D_ and mucosal nAb concentration (see S14 Fig). (C) Estimates for the probability that a single infectious virion starts a host infection, ψ, shown for each of the four individual macaque studies. The different symbols and bars depict the lowest, mean and highest values of ψ, based on the different estimates for the number of potentially infectious virions as shown in (B). For this analysis, only immunization and challenge regimes resulting in infection of test animals were considered. The average value of ψ (1.65x10^-5^) across all studies is indicated.

We next predicted nAb neutralization efficacy and host infection probability *in vivo* by re-assessing data from four studies of rhesus macaque vaginal virus challenge following passive immunization with nAbs b12 [8], 2G12 [7], PGT121 [10] and PGT126 [16]. Our approach consists of two parts: first, we utilized the nAb neutralization prediction model developed above (Fig 4) to estimate how many virions of the challenge dose are neutralized *in vivo* in dependence on the nAb immunization regime. Secondly, we connected this number of non-neutralized virions to the number of animals protected or infected for a given immunization and challenge regime. Ultimately, this allows us to derive the probability for a single infectious virion to establish a host infection (Fig 5A).

This modelling of HIV-1 neutralization *in vivo* following vaginal challenge requires data on vaginal nAb concentrations and nAb K_D_ as well as the virus-specific parameters of inoculum size, $\bar{\eta }$, T and N. Importantly, we also require the probability of virions to penetrate mucosal epithelial layers to come in contact with target cells (p_pen_); this parameter was recently estimated elegantly by Carias *et al*. [50]. The four selected macaque studies reported the majority of nAb and virus-specific parameters (S6 and S7 Tables), allowing us to employ our modelling approach in a post-hoc analysis. Essential data of the four analyzed studies are listed below:

(i) In the b12 study [8], 5 animals were immunized weekly with 1 mg/kg nAb b12, giving average serum b12 concentrations of 41.8 μg/ml and extrapolated vaginal mucosal concentrations of 0.47 μg/ml (S6 and S7 Tables). Animals were challenged bi-weekly with virus SHIV-P3, first 11 times with 3 TCID50 (397.500 virions) and subsequently 40 times with 10 TCID50 (1.325.000 virions). One animal was infected after 6 challenges with 3 TCID50, while 3 animals were infected after 6, 23 and 38 additional challenges with 10 TCID50, and one animal remained uninfected throughout the challenge regime.
(ii) In the 2G12 study [7], 5 animals were immunized once with 40 mg/kg nAb 2G12, giving average serum 2G12 concentrations of 1053 μg/ml and extrapolated vaginal mucosal concentrations of 12 μg/ml (S6 and S7 Tables). Animals were challenged once with 500 TCID50 (66.250.000 virions) of virus SHIV-P3. Three animals were protected, while 2 became infected.
(iii) In the PGT121 study [10], 5 animals per group were immunized once with either 5, 1 or 0.2 mg/kg of nAb PGT121, giving average serum PGT121 concentrations of 95, 15 and 1.8 μg/ml, respectively. Vaginal mucosal concentrations were measured at 0.9 and 0.2 μg/ml for the 5 and 1 mg/kg immunizations and extrapolated at 0.02 μg/ml for the 0.2 mg/kg immunization (S6 and S7 Tables). Animals were challenged once with 300 TCID50 (39.750.000 virions) of virus SHIV-P3. All animals with 5 and 1 mg/kg PGT121 immunizations were protected, while 3 animals were protected and 2 became infected for the 0.2 mg/kg immunization.
(iv) In the PGT126 study [16], 5 animals per group were immunized once with either 10, 2 or 0.4 mg/kg of nAb PGT126, giving average serum PGT126 concentrations of 98, 20 and 3.6 μg/ml, respectively. Vaginal mucosal concentrations were extrapolated at 1.1, 0.22 and 0.04 μg/ml for the 10, 2 and 0.4 mg/kg immunizations, respectively (S6 and S7 Tables). Animals were challenged once with 300 TCID50 (39.750.000 virions) of virus SHIV-P3. All animals with 10 mg/kg PGT126 immunizations were protected, while 2 animals were protected and 3 became infected for the 2 mg/kg immunization, and 1 animal was protected and 4 became infected for the 0.4 mg/kg immunization.

To derive nAb K_D_ data for the challenge virus Env P3 used in all four studies, we utilized the known IC50 for 2G12, b12, PGT121 and PGT126 against P3 and inferred the corresponding K_D_ values by linear regression (S6 and S7 Tables). We further assumed that virus strain P3 has T = 2 and $\bar{\eta }=20$ as previously determined for the closely related strain P3N (S3 Table). These data enabled us to predict neutralization curves for the four nAbs against SHIV-P3 (S14 Fig). We then superimposed these nAb neutralization curves with the estimated nAb concentrations in the vaginal mucosa at the time of challenge. In this analysis, we allowed both nAb K_D_ and mucosal nAb concentrations to vary 2-fold around the estimated values to account for potential inaccuracies in the extrapolation procedures. This analysis yielded windows for the extent of SHIV-P3 inoculum neutralization by the four nAbs *in vivo* (S14A Fig, colored parts of the neutralization curves). We predicted almost complete SHIV-P3 inoculum neutralization for the highest PGT121 and PGT126 immunization regimes, while the b12 and 2G12 immunizations and the low doses of PGT121 and PGT126 immunizations yielded intermediate SHIV-P3 neutralization levels, mirroring the protective effects seen in the respective challenge studies [7, 8, 10, 16].

Importantly, our model also allowed us to determine the probability that a single infectious virion starts a host infection. To this end, we first calculated the fraction of SHIV-P3 virions that remained potentially infectious in the four macaque challenge studies, i.e. virions with at least T non-neutralized trimers (S14B Fig). Multiplying these fractions of non-neutralized virions with the total number of virions in the respective challenge inocula provided an estimate of the number of virions that remained potentially able to infect each animal (Fig 5B).

To investigate how this number of non-neutralized, infectious virions translates into systemic host infection we utilized a further parameter. During vaginal challenge not all virions of the inoculum will penetrate the vaginal epithelial layers to come in contact with mucosal CD4^+^ target cells; the probability of epithelial penetration is given by p_pen_. We assumed that only 0.235% of virions will penetrate the genital tract epithelium as experimentally estimated [50]. Based on this, we derived the number of infectious virions in the four challenge studies that can potentially contact mucosal target cells and set these number in relation to the observed infection outcomes (Fig 5A). This delivered the probability for a single infectious virion to establish a systemic host infection, denoted ψ (Fig 5C). Intriguingly, we obtained closely matching ψ-estimates for the four independent macaque studies, with an average value of $\hat{\psi }=1.65\times 10^{-5}$.

Next, we asked whether a similar analysis would be possible using *in vitro* nAb neutralization data (i.e., nAb IC50 and Hill parameter). For this analysis we utilized *in vitro* neutralization data of SHIV-P3 with nAbs PGT121, PGT126 and b12 (S11B Fig) and analyzed the respective macaque challenge studies. We obtained a closely matching estimate of ψ, i.e. $\hat{\psi }=2.95\times 10^{-5}$ (S15 Fig) as with our mechanistic population neutralization model (Fig 5C). Based on these two complementary analyses, we conclude that only one in ~30.000 to 60.000 infectious virions that have penetrated the vaginal epithelial layers will succeed in systemically infecting the host. This estimate thereby provides a quantitative evaluation of the bottlenecks encountered by HIV-1 during transmission in the genital mucosa.

### Predicting human infection probabilities and nAb levels required to protect from HIV-1 acquisition

We next used the estimate of HIV-1 virion host infection probability ($\hat{\psi }=1.65\times 10^{-5}$) to predict male to female HIV-1 transmission risk per sex act and nAb protection efficacy. In this analysis, we define the per-act HIV-1 inoculum as the number of HIV-1 virions per ejaculate; this number is given by per-ejaculate semen volume and semen viral load. We then assumed that only a fraction of virions in the inoculum will penetrate the vaginal epithelial layers, defined by p_pen_ [50]. We next determined the number of virions that are potentially infectious, i.e. have at least T trimers, and multiplied the fraction of penetrating infectious virions with the probability for each infectious virion to initiate host infection, ψ. Thus, we obtained the probability of host infection in dependence on HIV-1 inoculum size. In addition, we modelled protective effects of nAbs present in the vaginal mucosa; here, the extent of HIV-1 inoculum neutralization (and hence the magnitude of protection) depends on mucosal nAb concentration and nAb binding affinity (K_D_) for the Env trimer.

**Fig 6:**
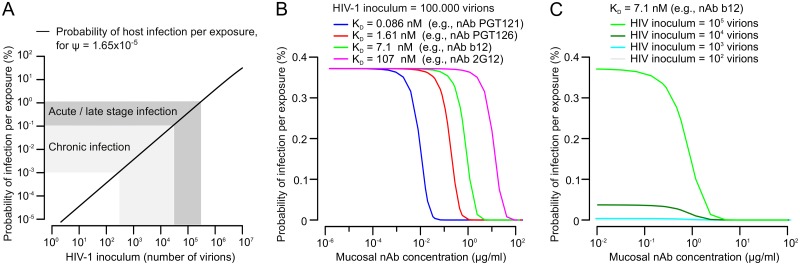
Predicting protective antibody levels in vaginal HIV-1 transmission. (A) Model predictions for male to female per-act HIV-1 transmission risk in absence of nAbs and in dependence of HIV-1 inoculum size. The range of typical HIV-1 inoculum sizes in semen (defined by semen viral load and semen volume) observed in chronic and acute stages of HIV-1 infection and the corresponding predicted per-act infection risks are indicated in grey shadings. (B) Model predictions for male to female per-act HIV-1 transmission risk in dependence on vaginal mucosal nAb concentrations. A relatively high HIV-1 inoculum of 100.000 virions was assumed. NAbs with four different K_D_ are modelled, spanning the range from less potent to very potent nAbs. (C) Model predictions for male to female per-act HIV-1 transmission risk in dependence on vaginal mucosal nAb concentrations, assuming four different HIV-1 inoculum sizes and a medium-potent nAb.

In a first step, we calculated infection probabilities of women during a single penile-vaginal intercourse in dependence on HIV-1 inoculum size, in absence of nAbs. We performed this analysis for HIV-1 virions with the entry characteristics of the transmitted-founder strain BG505 ($\bar{\eta }=9.5$ trimers per virion, T = 2 entry stoichiometry; S3 Table). The resulting relation between HIV-1 inoculum size and per-act female host infection probabilities is shown in Fig 6A.

This relation needs to be interpreted with regard to empirical data of human HIV-1 semen viral loads and per-act transmission risk. In chronic HIV-1 infection, semen viral load typically ranges between ≤ 200 and 20.000 HIV-1 RNA genome copies per mL seminal plasma (i.e., ≤ 100 to 10.000 virions / mL, assuming two viral genomic RNA copies per virion). During acute infection, semen viral load typically ranges between 20.000 and 200.000 RNA copies / mL (10.000 to 100.000 virions / mL) [33]. In rare cases, semen viral loads of several million RNA copies / mL (>1.000.000 virions / mL) were reported [51]. Assuming an average per-ejaculate semen volume of 3 mL [51, 52], this results in typical HIV-1 inoculum sizes of ≤ 300 to 30.000 virions during chronic HIV-1 infection, 30.000 to 300.000 virions during acute infection, and > 3.000.000 virions in rare cases. The corresponding per-act female infection probabilities predicted by our model are ≤ 0.001 to 0.11% during chronic infection (one in ≥ 87.000 to one in 876 sexual contacts), 0.11 to 1.14% during acute infection (one in 876 to one in 88 sexual contacts), and maximum values exceeding 11% (one in 9 sexual contacts) (Fig 6A).

How do these estimates compare to empirical data on female per-act infection probabilities? A frequently stated number for male-to-female penile-vaginal transmission risk is 1 in 1000 sexual contacts (0.1%). However, as previously discussed [32, 53, 54], this likely represents a lower bound of per-act infection risk and may be dangerously misleading. Our model predicts that an HIV-1 inoculum of ~26.000 virions (i.e., a semen viral load of ~17.000 RNA copies per mL, assuming 3 mL semen volume) would result in a female per-act infection risk of 0.1%. This semen viral load lies within the range typically observed during chronic HIV-1 infection. In contrast, several studies reported per-act penile-vaginal female infection risks between 0.5 and 10%, presumably linked to acute or late-stage HIV-1 infection of the male partner and correspondingly higher semen viral loads ([32, 53, 54] and references therein). Indeed, our model predicts HIV-1 inoculums of 130.000 to 2.770.000 virions (semen viral loads of 87.000 to 1.850.000 HIV-1 RNA copies / mL) to result in per-act infection risks of 0.5 to 10%. These values represent the range of semen viral loads typically observed during acute HIV-1 infection and rare cases of exceedingly high semen viral load. Thus, our model appears to precisely capture and recapitulate the interplay between HIV-1 inoculum size and infection probability during HIV-1 transmission in the female genital tract suggested by empirical studies.

Having thus obtained good indications that our model and the decisive parameter of ψ reliably mirror empirical data of human mucosal HIV-1 transmission, we next aimed to model the protective effects of nAbs present in the vaginal mucosa. First, we assumed an HIV-1 inoculum of 100.000 virions and modelled neutralization by four nAbs with a broad range of K_D_ (Fig 6B). Not surprisingly, this showed that with increasing nAb K_D_ (decreased Env trimer binding affinity), higher mucosal nAb concentrations are required to provide protection from infection. Specifically, we found that nAbs with a high Env binding affinity, such as PGT121, would afford protection from infection well below mucosal nAb concentrations of 0.1 μg/mL. This suggests that nAbs with high Env binding affinity could exert protective effects *in vivo* at relatively low doses.

We next looked more closely at the protective effects of a nAb with intermediate Env affinity and reduced potency, reasoning that such nAbs may be more readily inducible by vaccination. We chose nAb b12 as an example and modelled its protective effects in the vaginal mucosa against various HIV-1 inoculum sizes (Fig 6C). This analysis showed that such medium-potent nAbs may provide considerable protective effects starting in the range of 1 μg/mL mucosal nAb concentration.

**Fig 7:**
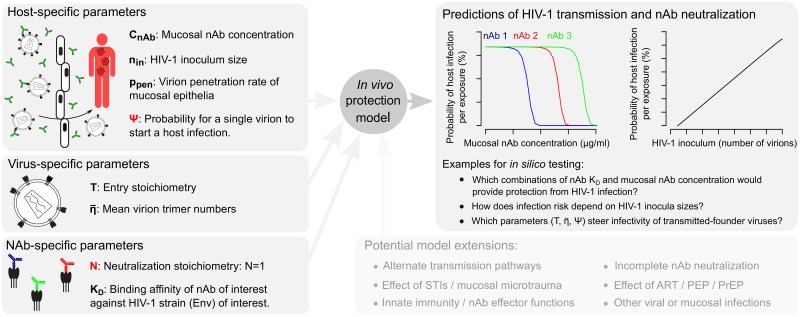
Modelling HIV-1 mucosal transmission and antibody neutralization. Summary of the parameters incorporated into our model (left), allowing *in silico* analysis of host HIV-1 infection probabilities and the *in vivo* protective effects of neutralizing antibodies (right). Parameters shown in red were estimated in this study, while parameters shown in black were retrieved from the literature. The model framework can be expanded by a range of parameters to investigate additional factors involved in HIV-1 transmission and prevention (bottom), and may serve as blueprint for similar efforts in other viral disease settings or mucosal infection processes.

In summary, the analyses shown in Fig 6 suggest that our model has the potential to retrieve information relevant to human mucosal HIV-1 transmission, offering opportunities for further model extension and application in HIV-1 vaccine research (Fig 7).

## Discussion

Broadly neutralizing antibodies are considered a crucial component of many vaccines or infectious disease therapeutics. For HIV-1, defining the best lead antibodies has proven complex and affords intensive *in vitro* and *in vivo* efficacy testing in both animal models and humans. Thus, *in silico* modelling approaches that support such trials would be of enormous value; however, establishment of such models depends on detailed mechanistic knowledge of HIV-1 transmission and nAb neutralization processes. Here, we provide a step towards this by presenting an experimental and mathematical analysis of HIV-1 nAb neutralization spanning from the molecular to the organismal level, providing highly relevant quantitative insights into the initial steps of mucosal HIV-1 infection and its inhibition by nAbs.

We first assessed the molecular interplay between nAbs and the HIV-1 Env trimer and confirmed that nAb occupancy of one Env subunit is sufficient for trimer neutralization (N = 1). It is important to note that N refers to the number of subunits within one trimer required to be nAb-bound for loss of functionality. This definition does not exclude the possibility that one nAb binds two adjacent trimers on the virion surface, which would result in lower nAb concentrations required for virion population neutralization.

N = 1 proved true for all nAbs tested here, irrespective of epitope specificity or nAb breadth and potency, including weakly neutralizing polycloncal IgG in HIV-1^+^ patient plasma (Figs 2 and 3). This indicates that the intricate machinery of the HIV-1 Env trimer, required to mediate binding to and fusion with the target cell membrane, is dependent on full functionality of all three trimer subunits. By confirming that HIV-1 neutralization follows an N = 1 stoichiometry, we defined a decisive numerical requirement for HIV-1 virion neutralization by nAbs. Indeed, in combination with the mean number of trimers per virion ($\bar{\eta }$) and the number of trimers required for entry (T), N defines the threshold number of antibodies required to neutralize a single HIV-1 virion or entire virion populations [27].

Alongside a range of additional parameters that were only recently determined (Fig 1), the estimation of N enabled us to use a mathematical model to analyze the interplay between nAbs, HIV-1 virion populations and the animal or human host during HIV-1 infection. We demonstrate the power of this model through several analyses. First, we predicted IC50 values of various nAbs and found that the predicted values closely matched empirical IC50 values (Fig 4). Next, we analyzed published data of macaque passive antibody immunization, vaginal virus challenge studies (Fig 5). We utilized the data presented in these studies to recapitulate virus inoculum neutralization by nAbs *in vivo* and to estimate the probability that a single infectious virion starts a productive host infection ($\hat{\psi }=1.65\times 10^{-5}$).

In a final set of analyses, we applied the estimate of ψ to model HIV-1 infection in the female genital tract and its neutralization by nAbs (Fig 6). We found that the per-act likelihood of female HIV-1 infection is clearly driven by the size of the virus inoculum, and we retrieved per-act virus transmission probabilities in good agreement with empirical estimates [32, 33, 51, 53]. Furthermore, we provide estimates for mucosal nAb concentrations required to provide protection from infection, indicating that nAb concentrations in the low μg/ml range may provide protection from mucosal HIV-1 transmission. Similar concentrations of HIV-1-specific IgG are readily detectable in the vaginal mucosa of women with chronic HIV-1 infection, raising the possibility that such vaginal IgG concentrations may be achievable by vaccination [55].

The analyses of female infection risk shown in Fig 6 represent the synthesis of all previous analyses (Figs 2 to 5), incorporating all modelling parameters (Figs 1C and 7). It will remain difficult if not impossible to precisely determine per-act HIV-1 inoculum sizes and infection outcomes in a human study population as well as the effect of nAbs thereon. The data we obtained here suggests that our mathematical framework has the potential to retrieve some of this much needed information by *in silico* modelling of *in vivo* HIV-1 infection and nAb neutralization. Given this potential relevance, in the following we discuss these estimates and the underlying model assumptions in more detail.

First, our model framework was built on data from macaque vaginal challenge studies. While host differences certainly exist, basic principles of mucosal HIV transmission in macaques and humans are similar [56, 57]. Penetration of mucosal barriers has been shown in both cases to be a rapid but inefficient process resulting in focal infection of few mucosal CD4^+^ cells, with productive host infection frequently ensuing from a single transmitted-founder virus [50, 58–60]. The probabilities we derive here for virion infectivity in macaque vaginal mucosal transmission should thus provide valuable insight into human infection processes.

Secondly, we would like to point out the importance of patient-to-patient variation, especially for the relation between HIV-1 inoculum size and host infection probability (Fig 6A). Of note, the per-patient and per-act HIV-1 inoculum size may differ widely based on variation in semen viral load and semen volume, which may both range over several orders of magnitude [33, 51, 52, 61, 62]. Our analyses support the hypothesis that HIV-1 transmission probability from an infected male partner with semen viral loads as typically observed in the chronic stage of HIV-1 infection is relatively low (≤ 0.1%), and that the HIV-1 pandemic may be primarily driven by transmissions occurring through high semen viral loads during acute or late-stage infections [33, 63].

Third, we focused our analysis on male to female HIV-1 infection, as it represents the most frequent pathway of human HIV-1 transmission [64]. However, our model is not *per se* limited to the setting of penile-vaginal transmission and can be adapted to capture rectal, mother-to-child or intravenous HIV-1 transmission. For example, many recent non-human primate studies tested passive nAb immunization followed by rectal virus challenge [11–13, 16, 18, 19]. The data presented in these studies could be leveraged by our model framework, as demonstrated here for vaginal challenge, to estimate ψ for rectal infection and subsequently test hypotheses for rectal HIV-1 transmission risk.

Fourth, mucosal nAb levels are challenging to measure and were not available in all macaque challenge experiments analysed here. Thus, we extrapolated mucosal nAb concentrations based on blood plasma nAb levels, using the ratio between plasma and mucosal nAb concentrations from studies where both parameters were experimentally determined. While this provided valuable estimates, precise measurement of mucosal nAb levels would be ideal for future studies building on our model framework.

Overall, we would like to note that our model should be viewed as a starting point to further investigate *in vivo* HIV-1 infection and nAb neutralization processes, as it focuses solely on virus-antibody interactions leaving additional factors, such as the mucosal milieu and nAb effector functions, not accounted for. The model can and should be fine-tuned by incorporation of additional parameters once they become known (Fig 7). Mucosal HIV-1 infection is incompletely understood and bottlenecks in transmission that may specifically select viral variants have not been specified. It remains debated whether HIV-1 transmitted-founder strains show distinct properties or whether transmission is purely stochastic [65–68]; our approach may help to shed light on this important question. Additionally, a range of factors are considered to influence mucosal HIV-1 transmission including epithelial micro-trauma, local inflammation, presence of other sexually transmitted infections, mucosal target cell availability, and innate immune defences [24, 69–71]. Reliably parametrizing these conditions will remain challenging but could add valuable information in forthcoming studies utilizing our model framework. Furthermore, our model currently does not include selected nAb features that may impact on neutralization efficacy, including the effect of neutralization plateaus [13, 49, 72], the contribution of Fc-mediated mechanisms [73–75], the effect of non-neutralizing Abs [76–79] and the role of IgA in HIV-1 inhibition [80–82]. Most importantly with respect to neutralization efficacy, information on nAb half-life and tissue distribution is only starting to emerge [83, 84]. In combination, these factors likely contribute substantially to inter-patient variation in susceptibility to HIV-1 infection, and it will be highly interesting to incorporate them in future model extensions.

By estimating the probability that an infectious HIV-1 virion establishes an infection (ψ), and by being able to predict the effect of mucosal nAbs on HIV-1 infection risk, our study occupies a sweet spot between HIV-1 mathematical epidemiology and virus dynamics studies. Modelling studies of the epidemiological spread of HIV-1 [85–87], on the one hand, are typically not accounting for the transmitted virus dose, but rather assume a fixed probability of transmission between donor and recipient upon encounter. While the probability of transmission is often stratified by cofactors (such as sex or age of donor or recipient, or set-point viral load in the donor) it lacks the detailed mechanistic underpinning that our approach provides. Virus dynamics studies of HIV-1, on the other hand, are mostly concerned with the virus dynamics once the infection has been established, often focusing on changes brought about by treatment [88, 89]. In these studies, the anatomy of the host is usually not considered in detail. The necessity of integrating the within-host dynamics into the epidemiological modelling of any pathogen has been theoretically conceived [90, 91]. In the context of HIV-1, however, such so-called nested or embedded approaches have so far been used only in theoretical studies on the evolutionary dynamics of HIV-1 [92–94]. In a few studies, the probability of establishment of an infection along with its potential modulators, such as microbicides, T cells, exposure history, or latency, has been investigated for HIV-1 and SIV [95–101] as well as for other pathogens [102]. However, these studies did not provide the bottom-up empirical link between the establishment of an infection and mucosal antibody levels that is central to our approach. A notable exception is the study by McKinley et al. [103] who presented a model for the early virus dynamics and the effect of antibodies. In contrast to our model, however, they predict neutralization success purely on the basis of the binding kinetics (K_D_) of antibodies to the HIV-1 Env trimer. In summary, our study thus provides a comprehensive set of essential and empirically-derived parameters for modelling efforts that aim to combine the within and between host dynamics of HIV-1 infection.

In conclusion, our combined experimental-mathematical approach delivers precise estimates of virion-antibody interaction stoichiometry, single-virion mucosal transmission probability, male to female per-act infection risk and *in vivo* nAb neutralization efficacy. These data represent novel quantitative insight into both the molecular details of HIV-1 antibody neutralization and the systemic level of mucosal HIV-1 infection. Our findings suggest that the model framework introduced here incorporates essential parameters that capture decisive steps of early HIV-1 infection and nAb neutralization, and thus provides means to predict and analyse the effects of nAbs on blocking mucosal virus transmission *in vivo*. Furthermore, our framework offers vast options for model extensions to investigate additional parameters or entirely different infection scenarios (Fig 7). Thus, our work represents a versatile, generalizable modelling tool to enhance our fundamental mechanistic knowledge of virus-antibody interactions and viral mucosal transmission, and can serve as stepping stone for planning and post-hoc evaluation of HIV-1 antibody-based treatment and vaccine trials.

## Materials and methods

### Ethics statement

Plasma samples from chronically infected individuals (ZA110, Pat117, Pat118, Pat122) were obtained from biobank samples previously collected during two approved clinical trials, the Swiss treatment interruption trial [104–108] and the Zurich Primary HIV-infection (ZPHI) study (ClinicalTrials.gov identifier NCT00537966) [109]. Written informed consent was obtained from all individuals in the respective studies according to the guidelines of the ethics committee of the canton Zurich.

### Cells and Env variants

293-T cells (obtained from the American Type Culture Collection) and TZM-bl cells [110] (obtained from the NIH AIDS Reagent Program) were cultured as described [111]. The origin of HIV Env plasmids is listed in S3 Table. Env point mutations were generated by site-directed mutagenesis (Agilent QuikChange II XL kit). All Env mutants were confirmed by sequencing. V1V2-deleted Envs were previously described [44]. The Luciferase reporter HIV-1 pseudotyping vector pNLLuc-AM was previously described [111].

### Reagents

MAbs (see S4 Table) were kindly provided by: PG9, PGT121, PGT128, PGT135, PGT145, b12 and b6 by Dr. Dennis Burton, The Scripps Research Institute, La Jolla, USA. 2F5 and 2G12 by Dr. Dietmar Katinger, Polymun Scientific, Vienna, Austria. 17b and 48D by Dr. James Robinson, Tulane University, New Orleans, USA. 447-52D was purchased from Polymun Scientific. Expression plasmids for VRC01 and PGV04 were provided by Dr. John Mascola, National Institutes of Allergy and Infectious Diseases, Bethesda, USA. Expression plasmids for NIH45.46 and 1.79 were provided by Dr. Michel Nussenzweig, The Rockefeller University, New York, USA. T-20 was purchased from Roche Pharmaceuticals.

### Estimating the stoichiometry of neutralization, N, with mixed trimer assays

To produce HIV-1 pseudovirus stocks expressing mixed trimers with varying ratios of nAb-sensitive to nAb-resistant Env, 293-T cells in 6-well plates (250.000 cells per well in 2 ml complete DMEM, seeded 24 h pre-transfection) were transfected with 3 μg pNLLuc-AM and 1 μg Env expression plasmids, using polyethyleneimine (PEI) as transfection reagent. The ratio of sensitive to resistant Env was varied to yield combinations with 100, 90, 70, 50, 30, 10 and 0% of resistant Env. After overnight incubation the transfection medium was replaced with 2.5 ml fresh complete DMEM and virus-containing supernatants were harvested 48 h post transfection. To determine virus infectivity, serial dilutions of virus stocks were added to TZM-bl cells in 96-well plates (10.000 cells per well) in DMEM supplemented with 10 μg/ml DEAE-Dextran. TZM-bl infection was quantified 48 h post-infection by measuring activity of the firefly luciferase reporter (in arbitrary relative light units, RLU). The neutralization activity of mAbs and patient plasma against the mixed trimer virus stocks was evaluated on TZM-bl cells as described [111]. Sufficiently high starting concentrations of inhibitors were chosen to yield clear neutralization plateaus, and data were fitted in GraphPad Prism version 7.0 using the sigmoidal dose-response (variable slope) function. In cases where no clear neutralization plateaus were obtained (less than two consecutive data points giving the same level of neutralization), the position of the expected plateau was provided by the curve fit. Subsequently, the relative infectivity (RI) of each virus stock under saturating inhibitor concentrations was calculated. The resulting RI values were plotted over the fraction of resistant Env (f_R_) of each virus stock and the data were analyzed with mathematical models.

### Env expression analysis by flow cytometry

293-T cells were transfected with Env and Rev plasmids and processed for flow cytometry as described [37]. Env on the cell surface was detected with biotinylated mAb 2G12 (5 μg/ml) and Streptavidin-APC (BioLegend, San Diego, USA; 1:400 dilution) or Abs 1.79 and PG9 (5 μg/ml) and Cy5-conjugated F(ab′)_2_ goat anti–human IgG (Jackson ImmunoResearch, West Grove, USA; 1:500 dilution) followed by cell analysis on a CyAN ADP flow cytometer (Beckman Coulter, Brea, USA).

### Mathematical modeling to estimate N and HIV-1 population neutralization

#### Trimer neutralization model

The trimer neutralization model is used to determine the stoichiometry of neutralization, N. This model was described in detail in Magnus and Regoes, 2010 [26]. Briefly, it predicts relative virus infectivity as a function of trimer composition. The model factors in the number of trimers needed for virus entry (the entry stoichiometry, T) and the distribution of trimer numbers across the virion population, η. Trimer assembly is modeled by randomly choosing three Env subunits from a subunit pool consisting of nAb-sensitive and nAb-resistant Envs (according to a binomial distribution). The fraction of nAb-resistant Env subunits is denoted by $\bar{\eta }$. As extensive determination of the trimer number distribution, η, is lacking, we use a discretized Beta distributed trimer number distribution with mean $\bar{\eta }$ and variance 49/14 $\bar{\eta }$ according to Magnus *et al*., 2009 [25] and Zhu *et al*., 2006 [112].

Thus, the relative infectivity (RI) is given by

$${\text{RI}}_{\text{N}}=\frac{\sum_{s=T}^{s_{\text{max}}}\eta_{s}\left(\sum_{g=T}^{s}\left({}_{g}^{s}\right)\alpha_{N}^{g}{\left(1-\alpha_{N}\right)}^{s-g}\right)}{\sum_{s=T}^{s_{\text{max}}}\eta_{s}}$$

where *s*_*max*_ = 100 is the maximal number of trimers per virion and $\alpha_{N}=\sum_{m=(3-N)+1}^{3}\left({}_{m}^{3}\right)f_{R}^{m}{\left(1-f_{R}\right)}^{3-m}$ is the probability that a trimer is functional, i.e. non-neutralized by nAb, which depends on N.

For estimating N, we use virus strain-specific estimates of T and $\bar{\eta }$ determined in Brandenberg et al., 2015 [28]. Note that virions with less than T trimers are considered as non-infectious. For example, assuming a mean trimer number of $\;\bar{\eta }=11.8$ and a variance of 41.3, only 99.36% of all virions are infectious if T = 2, respectively 78.23% for T = 7 (S16A Fig). Broader trimer number distributions, i.e. distributions with higher variance, result in lower fractions of infectious virions (S16B Fig). These numbers are not equal to the infectious to non-infectious virion ratios, which are normally much smaller. The reason for this is that in the infectious to non-infectious ratio one usually compares the virions actually infecting a cell to those that do not. In our model we approximate this by summing over the infectious virions given a certain fraction of mutated Envs and by subsequently dividing this by the infectious virions of a completely nAb-susceptible virus strain (see the RI-equation above).

Note that N is defined as the minimal number of trimer subunits required to be bound to an antibody such that the trimer loses functionality. Following this definition, N can only take the values 1, 2 or 3. In so-called soft threshold models [26, 29], binding of each antibody incrementally reduces the probability of the trimer to take part in infection. These models can potentially be interpreted as N<1. To test the soft threshold model, we use the same methods on our RI data sets as previously described in [26].

In addition to the above-described model, we developed a model in which a trimer has three potential epitopes of which only one can be bound by an antibody. Binding of the first antibody disallows further antibody binding. This model reflects the binding behavior of PG9 and PGT145-like antibodies that are known to only bind with one antibody per trimer.

The RI curve is fitted to the experimental data with a nonlinear regression method (residual sum of squares, rss) and the estimate of N is the N-value for which the rss is the lowest. In addition, we performed a bootstrap procedure with 1000 replicates to determine the confidence/reliability of the N estimates. All estimates have a confidence of at least 95%. For a sensitivity analysis we estimate N for different T’s ranging from 1 to 20 and the mean number of trimers $\bar{\eta }$ ranging between 5 and 25 (see S4 Fig). Given the experimentally determined T and $\bar{\eta }$ values [28], our estimates for N are those with the lowest rss-values. If we leave $\bar{\eta }$ and T free, however, the minimal rss values would be reached for a combination of lower mean virion trimer numbers $\bar{\eta }$ in combination with higher stoichiometries of entry, T (see Fig 2E).

### Population neutralization model

(i) NAb concentrations that confer sterilizing immunity

To tackle the question how high nAb *concentrations* must be to neutralize a given virion population, we first study the *number* of nAbs required to perform this task. We start with a virus population with *n*_*v*_ virions. Each virion has a random trimer number *S*_*i*_ that follows a discretized Beta distribution with mean $\bar{\eta }$ and variance $v=49/14\times \bar{\eta }$ (see above). We let nAbs bind to these virions until all virions are neutralized. Neutralization of virion *i* is achieved when at least (*S*_*i*_ − *T* + 1) trimers are bound to at least *N* nAbs. This simulation is repeated *n*_*r*_ = 1000 times and the mean of the nAb numbers to reach neutralization is calculated. We introduced this procedure in Magnus and Regoes, 2011 [27].

To transition from nAb numbers required for virion neutralization to nAb concentrations, we model the binding of a nAb, *Ab*, to an envelope protein, *E*, with a chemical binding equation [35]:

$$E+Ab\overset{k_{a}}{\underset{k_{d}}{⇌}}EAb$$

where *k*_*d*_ is the off-rate constant and *k*_*a*_ the on-rate constant. Assuming a first order reaction, the quotient of the product of the reactant concentrations divided by the product concentration follows:

$$K_{D}≔\frac{k_{d}}{k_{a}}=\;\frac{c\left(E\right)c\left(Ab\right)}{c\left(EAb\right)}$$

The fraction of bound envelope proteins, *f*_*b*_, when the equilibrium is reached can then be calculated by

$$f_{b}=\;\frac{c\left(EAb\right)}{c\left(E\right)+c\left(EAb\right)}={\left(\frac{c\left(E\right)}{c\left(EAb\right)}+1\right)}^{-1}=\;{\left(\frac{K_{D}}{c\left(Ab\right)}+1\right)}^{-1}$$

This equation can be transformed to

$$c\left(Ab\right)=\;\frac{K_{D}f_{b}}{1-f_{b}}$$

With this equation it is possible to determine the nAb concentrations needed for sterilizing neutralization by determining the fraction of bound envelope proteins with the simulation tool described above.

(ii) Calculating the neutralization levels for different nAb concentrations

We start with calculating the percentage of neutralized virions when *n*_*Ab*_ nAbs are bound to *n*_*v*_ virions. Each virion has a discretized Beta distributed trimer number, *S*_*i*_, with mean $\bar{\eta }$ and variance $v=49/14\times \bar{\eta }$. We then distribute the *n*_*Ab*_ nAbs to the virions such that each envelope protein has the same probability of being bound. Thus *A*_*i*_ nAbs bind to virion *i*.

The probability that a virion with *s* trimers is neutralized when *a* nAbs bind to the complete virion can be calculated as follows:

P(neut|S=s,A=a)=0 if a<(s−T+1)N; P(neut|S=s,A=a)=1 if a≥3(s−T)+(N−1)T+1

and

$$P\left(neut|S=s,A=a\right)=\sum_{\left(y_{1},\;y_{2},y_{3}\right)\in \varepsilon_{a,s}}P\left(Y_{1}=y_{1},\;Y_{2}=y_{2},\;Y_{3}=y_{3}\right)$$

where *P*(*Y*_1_ = *y*_1_, *Y*_2_ = *y*_2_, *Y*_3_ = *y*_3_) is the probability that *y*_*j*_ trimers are bound to *j* nAbs and ℇ_*a*, *s*_ is the set of all combinations of *a* nAbs to *s* trimers such that at least (*s* − *T* + 1) trimers are bound to at least *N* nAbs. For *N* = 1 this set is

$$\varepsilon_{a,s}=\text{(}\left(y_{1},y_{2},y_{3}\right)\in {\mathbb{N}}_{0}^{3}|y_{1}=m,y_{2}=3\zeta -a-2m,y_{3}=a+m-2\zeta \;with\;0\leq m\leq \text{min}\left(s,a\right)\;and\;s-T+1\leq \zeta \leq s)$$

The fraction of neutralized virions, $f_{n_{v}}$, can thus be calculated by:

$$f_{n_{v}}=\frac{1}{n_{v}}\sum_{i=1}^{n_{v}}P\left(neut|S_{i}=s_{i},A_{i}=a_{i}\right)$$

To calculate the mean fraction of neutralized virions, the above described procedure is performed *n*_*r*_ = 1000 times.

Several nAbs have been shown to bind with only one antibody per trimer, including nAbs PG9, PG16 and PGT145 [38, 47, 49]. To account for this binding behavior, we extended the above described model. We still assume that each trimer has three epitopes. However, as soon as one nAb binds to a trimer, no additional nAbs can bind. The fraction of bound epitopes is then *f*_*b*, *epitopes*_ = 1/3*f*_*b*, *trimers*_ and a virion with *s* trimers is neutralized when (*s* − *T* + 1) trimers are bound by one nAb.

#### *In vivo* protection model

Here, we use the above described model to predict virus neutralization levels in macaque passive nAb immunization, vaginal challenge studies [7, 8, 10, 16]. In Fig 5, we show neutralization curves predicted with the above described models based on estimated nAb K_D_ values for the P3 challenge virus. The results are based on simulations with *n*_*v*_ = 1000 virions.

#### Determining the probability that an infectious virion starts a host infection

How high is the probability, here denoted ψ, that an infectious virion starts a host infection? This probability is modeled to be independent of the number of antibodies bound as long as the virion is still infectious (i.e., has at least T functional trimers). With our stoichiometric framework and the results from the macaque challenge studies we can approach this question. To this end, we derive a mathematical expression for the probability that infection happens given a certain HIV-1 inoculum size. We denote the number of virions forming the inoculum by *n*. Only virions that have penetrated the epithelial layer (happening with probability *p*_*pen*_), are not neutralized by antibodies (happening with probability 1 − *f*_*neut*_) and have at least T trimers on their surface (happening with probability $\sum_{k=T}^{s_{\text{max}}}\eta_{k}$) can end up productively infecting the host.

Thus, the effective inoculum size, *v*, is:

$$v=np_{pen}(1-f_{neut})\overset{s_{\text{max}}}{\underset{k=T}{\sum }}\eta_{k}$$

Let *Y*_*i*_, *i* = 1,…,*v*, be independent, identically distributed random variables with *Y*_*i*_ = 1 in case the i^th^ virion starts the infection and 0 else. The probability that a host infection starts is then

$$\begin{array}{l} \pi =1-P(\forall_{i=1,\ldots ,v}\;Y_{i}=0)\\ =1-\prod_{i=1}^{v}P(Y_{i}=0)\\ =1-P{\left(Y_{1}=0\right)}^{v}\\ =1-{\left(1-P(Y_{1}=1)\right)}^{v} \end{array}$$

As *ψ* = *P*(*Y*_1_ = 1) we can use the probability that an infection starts upon challenge, π, to estimate this parameter. To this end, we use the outcomes of the macaque challenge studies. However, the two types of challenges–repeated low dose versus single high dose–must be treated separately: (i) In single high dose challenge studies [7, 10, 16], the outcome of how many animals are infected follows a binomial distribution. (ii) In repeated low dose challenge studies [8], the number of challenges required to infect follows a geometric distribution.

*(i) Single high dose challenge studies*: We denote the number of animals included in a study with *m*. The number of infected animals, *Z*, follows a binomial distribution with infection probability *π*, i.e.
  $$P(Z=k)=\left({}_{k}^{m}\right)\pi^{k}{\left(1-\pi \right)}^{m-k}$$
  With *π* = 1 −(1−*ψ*)^*v*^ one can easily show that the maximum likelihood estimator for *ψ* is
  $$\hat{\psi }=1-{\left(1-\frac{k}{m}\right)}^{1/v}$$
*(ii) Repeated low dose challenge study*: In the low dose challenge study, the animals were first challenged with an inoculum of *n*_*1*_ = 397500 virions 11 times successively. The animals that were not infected in this phase were then successively challenged with *n*_*2*_ = 1325000 virions. The number of infectious virions in phase 1, *v*_*1*_, and in phase 2, *v*_*2*_, can be calculated by taking into account the penetration probability, *p*_*pen*_, the fraction of neutralized virions, *f*_*neut*_, and the probability of being infectious. Thus,
  $$\begin{array}{l} v_{1}=n_{1}p_{pen}(1-f_{neut})\sum_{k=T}^{s_{\text{max}}}\eta_{k}\\ v_{2}=n_{2}p_{pen}(1-f_{neut})\sum_{k=T}^{s_{\text{max}}}\eta_{k} \end{array}$$

We define ψ as the probability that one infectious virion starts a host infection. The probability that an infection is started upon one challenge in phase 1, *π*_*1*_, and in phase 2, *π*_*2*_, is then

$$\begin{array}{l} \pi_{1}=1-{\left(1-\psi \right)}^{v_{1}}\\ \pi_{2}=1-{\left(1-\psi \right)}^{v_{2}} \end{array}$$

For the likelihood function, *L*(*ψ*), describing the entire low dose challenge experiment, we need to factor in all geometrical distributed waiting times for each animal. One animal was infected after six challenges with viral load *n*_1_. Four animals were challenged eleven times with viral load *n*_1_ but remained uninfected. They were then challenged with viral load *n*_2_. Three animals got infected after 6, 23, and 28 challenges with viral load *n*_2_. One animal remained uninfected even after 40 challenges with viral load *n*_2_. Thus:

$$\begin{array}{l} L(\psi )={\left(1-\pi_{1}\right)}^{5}\pi_{1}\\ \times {\left(1-\pi_{1}\right)}^{11}{\left(1-\pi_{2}\right)}^{5}\pi_{2}\\ \times {\left(1-\pi_{1}\right)}^{11}{\left(1-\pi_{2}\right)}^{22}\pi_{2}\\ \times {\left(1-\pi_{1}\right)}^{11}{\left(1-\pi_{2}\right)}^{37}\pi_{2}\\ \times {\left(1-\pi_{1}\right)}^{11}{\left(1-\pi_{2}\right)}^{40}\\ =\pi_{1}\pi_{2}^{3}{\left(1-\pi_{1}\right)}^{49}{\left(1-\pi_{2}\right)}^{104} \end{array}$$

This function cannot be solved analytically in respect to ψ. We determine the maximum of the likelihood function numerically.

#### Estimating ψ

We used the above derived estimators to determine the probability that an infectious virion starts a host infection, ψ, based on the four macaque challenge studies [7, 8, 10, 16]. Due to the lack of K_D_ measurements for the binding of nAbs b12, 2G12, PGT121 and PGT126 to the challenge Env P3, we estimated these values based on the respective IC50 and the correlation between IC50 and K_D_ as demonstrated for BG505 (Fig 4, S6 and S7 Tables). Specifically, we calculated nAb IC50 values for different K_D_ values assuming a P3-specific trimer number distribution and entry stoichiometry. We then calculated the regression slope parameter between IC50 and K_D_ values and used this to extrapolate K_D_ values from measured IC50 values for the nAbs used in the macaque challenge studies.

In addition, the mucosal nAb concentrations were also extrapolated (S7 Table). Therefore, we estimated ψ for the extrapolated K_D_ and mucosal nAb concentrations and introduced a 2-fold variation of these parameters to account for the extrapolation procedure (Fig 5).

To probe whether it is sufficient to use *in vitro* nAb neutralization data to estimate *ψ*, we used the Hill-curve parameters to calculate the fraction of neutralized virions as a function of the antibody concentration $f_{neut}=c_{Ab}^{m}/\left(c_{Ab}^{m}+IC50^{m}\right)$ and plugged this into the equations for *v*_*1*_ and *v*_*2*_ above.

#### Predicting protecting antibody levels against HIV-1 infection

Using the estimated probability that an infectious virion starts a host infection, ψ, it is possible to predict the per-challenge infection probability as a function of the vaginal mucosal nAb concentration. The inoculum size in penile-vaginal transmission is the product of semen volume and the semen viral load. We directly calculate the probability of female infection upon one challenge with a fixed inoculum of size *n*. Following the notation in this paper, T is the stoichiometry of virus entry and $\bar{\eta }$ the virion trimer number distribution. The fraction of neutralized virions, *f*_*neut*_, depending on the nAb concentration is determined as described above. The probability that one challenge with an HIV-1 inoculum of size *n* leads to infection is thus:

$$P(\text{infection upon exposure})=1-{\left(1-\psi \right)}^{v}=1-{\left(1-\psi \right)}^{np_{pen}(1-f_{neut})\sum_{k=T}^{s_{max}}\eta_{k}}$$

In this equation, *v* is the number of virions of the inoculum that penetrated the epithelium, were not neutralized by nAbs and have sufficient trimers to potentially infect a host cell.

## Supporting information

S1 Fig Verification of Env antibody escape mutants. Our approach to estimate N relies on Env variants that are resistant or sensitive against neutralization by a given antibody (Fig 2A). Shown here are neutralization assays on TZM-bl cells with pseudotyped HIV-1 stocks carrying either wildtype (wt) or nAb escape or sensitivity mutant Envs as listed in S2 Table. Env variants and inhibitors tested are indicated above each panel. Only mutants showing complete escape from neutralization by the nAb of interest, or substantial loss in neutralization (<10% neutralization at a nAb concentration that results in 100% neutralization of the sensitive Env) were selected for further studies and are shown here. Data depict mean and SD from 2 independent experiments.
(PDF)

S2 FigInfluence of T and $\bar{\eta }$ on estimation of N with mixed trimer assays.(A) Model predictions for the influence of $\bar{\eta }$ on RI curve fits. (B) Mixed trimer assays with JR-FL wt ($\bar{\eta }=11.8$) and JR-FL ΔCT ($\bar{\eta }=19.4$) and nAb 2F5. The observed RI curve shifts are in agreement with shifts predicted by our model. (C) Data analysis revealed N = 1 for JR-FL ΔCT and nAb 2F5. (D) Model predictions for the influence of T on RI curve fits, assuming N = 1. (E) Mixed trimer assays with Envs JR-FL (T = 2) and NL4-3 (T = 7) and nAb 2F5. The observed RI curve shifts are in agreement with shifts predicted by our model. (F) Data analysis indicated N = 1 for NL4-3 and nAb 2F5. (G) and (H) To test the effect of mono versus bivalent nAb binding we compared 2F5 IgG and Fab fragment on strains JR-FL and NL4-3. Identical RI profiles for IgG and Fab fragments were observed. (I) Analysis of the JR-FL and NL4-3 2F5 Fab data indicated N = 1 in both cases.(PDF)

S3 Fig Influence of Env infectivity on mixed trimer pseudovirus neutralization assays. (A) Virus infectivity of JR-FL wt and indicated Env mutants, relative to JR-FL wt. D664N is a nAb 2F5 resistance mutation. (B) Ratio assays with nAb 2F5 and different combinations of JR-FL mixed trimer pseudovirus stocks. Combining JR-FL wt with JR-FL D664N, which have equal infectivity, yields a sigmoidal RI profile with an estimated N = 1 neutralization stoichiometry (Fig 2B and 2C). When the resistant Env (JR-FL D664N) is much more infectious than the sensitive Env (JR-FL ΔV1V2), the RI curve shifts strongly to the left (red squares). When the resistant Env (JR-FL ΔV1V2 D664N) is much less infectious than the sensitive Env (JR-FL wt), the RI curve shifts strongly to the right (blue triangles). We observed the same effects for HIV-1 strain NL4-3 (C and D). Thus, we only employed sensitive-resistant Env combinations with similar infectivities (within a two-fold range) for mathematical estimations of N. Interestingly, Env infectivity differences are not necessarily caused by expression differences (E and F). Expression of Envs on transfected 293-T cells was assessed by flow cytometry. (E) 293-T cells were transfected with the indicated ratios of JR-CSF wt and JR-CSF N160K, the latter being resistant to nAb PG9 and showing only 8% of JR-CSF wt infectivity (S2 Table). Both Envs are bound (and neutralized) equally well by nAb 2G12. The transfected 293-T cells were stained with both nAbs PG9 and 2G12 and analyzed by flow cytometry; the mean fluorescence intensity of each cell population in relation to cells expressing JR-CSF wt Env only are shown. Both Envs express to equal levels as judged by 2G12 staining. In addition, we observed a linear relation of nAb PG9 binding to cells in dependence on the ratio between PG9-sensitive and resistant Env, as expected. Thus, the low infectivity of JR-CSF N160K is not due to expression defects. (F) Identical analysis as in (E) for JR-FL wt and JR-FL ΔV1V2, the latter being highly neutralization sensitive and showing only 4% of JR-FL wt infectivity (panel A, S2 Table). Again, we observed equal expression levels of the two Envs as judged by 2G12 binding and a linear binding pattern for the V3 loop directed nAb 1.79, which potently neutralizes JR-FL ΔV1V2 but not JR-FL wt.
(PDF)

S4 FigRobustness analyses of N estimates against variation in T and $\bar{\eta }$.Shown are robustness analyses for the estimations of N shown in Fig 3A–3G. Each plot depicts the T and $\bar{\eta }$ values assumed for the analysis of each individual Env combination (white dots; two dots are shown in case of divergent estimates of T for a given Env, see S3 Table). The resulting estimates of N in dependence of T and $\bar{\eta }$ are color-coded: blue represents estimates of N = 1, green indicates estimates of N = 2, and red indicates estimates of N = 3. As shown, all N estimates are clearly within the N = 1 range.(PDF)

S5 Fig Bootstrap analyses for estimating N. Bootstrap analysis with 1000 replicates of all data shown in Fig 3, indicating that the N = 1 estimate is accurate.
(PDF)

S6 FigGoodness-of-fit plots for estimating N.Goodness-of-fit analyses of all data shown in Fig 3. As shown in Fig 2E, better fits would, in most cases, be obtained for lower values of T and $\bar{\eta }$.(PDF)

S7 Fig Analysis of the RI data (Figs 2 and 3) employing a soft threshold model. This model extension allows for partial trimer functionality loss upon antibody binding: the functionality of a trimer with one subunit bound to an antibody is π_1_, the functionality of a trimer with two bound subunits is π_2_, and a trimer with three subunits completely loses its functionality. In addition, the ability of a virion to infect a cell scales with the number of functional trimers, *g*, according to the equation *g*^*h*^/(*g*^*h*^ +T_1/2_^*h*^). (A) Estimates of trimer functionality upon antibody binding. For most virus-nAb combinations, the trimer loses functionality upon binding of one antibody. (B) Estimates for the steepness of the infection curve, *h*, and (C) estimates for the half maximal trimer number, *T*_1/2_. A bootstrap procedure shows very high uncertainty in these point estimates. We therefore discarded this model extension in favor of a “hard” threshold model.
(PDF)

S8 Fig Analysis of N on mixed trimer HIV-1 pseudovirus stocks with multiple nAb escape mutations. (A) to (C): Envs with multiple nAb resistance mutations allowed parallel assessment of various nAbs on the same set of mixed trimer virus stocks. The employed resistant and sensitive Envs and nAbs tested are indicated above each panel. The three mixed trimer setups shown here were not infectivity-matched, excluding mathematical estimation of N. However, inclusion of nAb 2F5, previously shown to neutralize with N = 1 (Fig 2), and graphic comparison of all RI curves indicates equal N (N = 1) of all nAbs tested.
(PDF)

S9 Fig Estimating N for the HIV-1 fusion inhibitor T-20. We utilized Env mixed trimer setups that allowed parallel assessment of both nAbs and the HIV-1 fusion inhibitor T-20 to obtain a direct comparison of the two inhibitor types. (A) Mixed trimer setup with Env mutants SF162 D279A and V549M N554D and nAbs NIH45.46, PGV04 and entry inhibitor T-20, indicating equal neutralization stoichiometry of N = 1. Note that in this setup the two Envs are reciprocally neutralization sensitive and resistant: SF162 D279A is resistant to nAbs PGV04 and NIH45.46 but sensitive to T-20; the opposite is true for SF162 V549M N554D. (B) Mixed trimer setup with Env mutants ZA110 ΔV1V2 and V549M N554D and nAbs b6, 447-52D and PGT145 and entry inhibitor T-20, indicating equal neutralization stoichiometry of N = 1. All graphs depict mean and SD from 2 independent experiments.
(PDF)

S10 FigPredicting antibody concentrations required for sterilizing neutralization of HIV-1 virion populations.(A) to (C) Predicted nAb concentrations required to completely neutralize HIV-1 virion populations as a function of varying size depending on (A) nAb K_D_, (B) HIV-1 entry stoichiometry, T, and (C) mean viron trimer number, $\bar{\eta }$. Panels (D) to (F) show the predicted nAb concentrations required to completely neutralize HIV-1 populations of 1, 141 or 9400 virions as a function of (D) nAb K_D_, (E) HIV-1 entry stoichiometry, T, or (F) the mean virion trimer number, $\bar{\eta }$. The required nAb concentration for complete neutralization is a complex function of T and $\bar{\eta }$, but linear in K_D_. This is the case because for a fixed viron population size, the fraction of envelope subunits required to be bound by nAb for complete neutralization, f_b_, is constant (see also Fig 4F).(PDF)

S11 FigComparison of predicted and experimental nAb neutralization curves.(A) Comparison of predicted and experimental nAb neutralization curves for HIV-1 strain BG505 across five nAbs. BG505-specific T, $\bar{\eta }$ and nAb K_D_ values (see S3 and S5 Tables) were used to predict the neutralization curves. Experimental data were obtained using BG505 pseudovirus stocks and TZM-bL reporter cells. We find that the predicted neutralization curves are notably steeper than experimentally obtained curves. (B) Comparison of predicted and experimental nAb neutralization curves for SHIV strain P3 across three nAbs. P3-specific T, $\bar{\eta }$ and nAb K_D_ values (see S3 Table) were used to predict the neutralization curves. Experimental data were obtained using replication-competent SHIV-P3 stocks and PBMC target cells, and were previously reported [10, 16]. We noted that especially for nAbs PGT121 and b12, predicted and experimental neutralization curves showed better agreement than for HIV-1 BG505 in (A).(PDF)

S12 FigThe trimer number distribution influences Hill coefficient.To follow up the discrepancy between experimental and predicted neutralization curve steepness (i.e., Hill coefficient; see S11A Fig) we asked which parameter of our model may steer the steepness of the predicted curves. We found that assuming a broader virion trimer number distribution, i.e. a higher variance in trimer numbers across virions, results in less steep predicted neutralization curves. For this graph we used the following parameters: $\bar{\eta }=9.5$, T = 2, N = 1, K_D_ = 8.65x10^-9^. Green: Var[η] = 1, black: Var[η] = 33.25, red: Var[η] = 100.(PDF)

S13 Fig Statistical model comparison between nAb IC50 predictions of our model and a simple K_D_-based model. We defined the nAb K_D_-based model as a linear model between the logarithm of the observed nAb IC50s and the logarithm of the nAb K_D_s. (A) Our model (red line, shown for T = 3) follows fluctuations in the mean nAb IC50s better (black dots, for the nAb data see S5 Table) than the K_D_-based model (green line). (B) Predicted nAb IC50 values plotted against the mean of the observed IC50 values are shown in green for the K_D_-based model and in red for our model. The visually better performance in predicting IC50 values of our model in comparison to a K_D_-based model is statistically confirmed by using the Akaike Information Criterion (AIC). We obtain lower AIC values for our model (AIC = 9.72 for our model with T = 2 and AIC = 8.7 for our model with T = 3) compared to an AIC = 11.7 for the K_D_-based model. Note that the value for 2G12 (around 5 nM K_D_) is not an outlier, as a Kolmogorov-Smirnov test for normality shows that the IC50 values are not distributed different from normal (p-value = 0.16).
(PDF)

S14 Fig Estimating SHIV-P3 inoculum neutralization in macaque challenge studies. (A) Predicted neutralization curves for nAbs b12, 2G12, PGT121 and PGT126 against the challenge virus SHIV-P3. Shaded areas indicate 2-fold variation in nAb K_D_. Dots depict measured or extrapolated vaginal nAb concentrations and the coloured areas indicate 2-fold variation in these nAb concentrations, indicating the SHIV-P3 predicted neutralization ranges achieved in the three studies. (B) The fraction of predicted non-neutralized SHIV-P3 virions in dependence on mucosal nAb concentrations, as shown in (A).
(PDF)

S15 FigEstimating ψ based on *in vitro* nAb neutralization data.Having obtained similar ψ estimates across the four analyzed macaque challenge studies using our mechanistic model (Fig 5), we asked whether similar results could be obtained using solely *in vitro* nAb neutralization data (thus bypassing the need to have estimates for T, $\bar{\eta }$ and nAb K_D_). We adjusted our model accordingly, requiring solely nAb IC50 and Hill coefficient as input. *In vitro* neutralization data of SHIV strain P3 with nAbs PGT121, PGT126 and b12 were previously reported [10, 16] and are shown in S11 Fig. Utilizing these data we obtained a closely matching ψ value of 2.95x10^-5^. We thus conclude that (i) the estimate of ψ is likely robust within the range of the two estimated values (i.e., between 1.65 and 2.95 x10^-5^), and (ii) in cases where information on T, $\bar{\eta }$ and nAb K_D_ are missing, *in vitro* nAb neutralization data may provide a good substitute to analyze macaque challenge studies as proposed herein.(PDF)

S16 FigRelation between virion trimer number distribution, T and viron infectivity.Shown are two hypothetical virion trimer number distributions, and how they influence the fraction of infectious virions in the population in dependence on T. (A) $\bar{\eta }=11.8$, Var[η] = 41.3. (B) $\bar{\eta }=11.8$, Var[η] = 600.(PDF)

S1 Table Parameters required for modelling HIV-1 virion population neutralization by antibodies. (DOCX)

S2 Table Env antibody escape or sensitivity mutants. (DOCX)

S3 Table Entry parameters and origins of HIV-1 strains. (DOCX)

S4 Table Antibodies employed in this study. (DOCX)

S5 Table Antibody IC50 and K_D_. (DOCX)

S6 Table Parameters of macaque passive immunization vaginal challenge studies. (DOCX)

S7 Table Extrapolation of vaginal antibody concentrations in macaque challenge studies. (DOCX)

S1 References Supporting references. (DOCX)
