# Supplementary figures and images for: Predicting HIV-1 transmission and antibody neutralization efficacy in vivo from stoichiometric parameters
Source: PLoS Pathog. 2017 May 4;13(5):e1006313. doi: 10.1371/journal.ppat.1006313 (PMC5417720; doi:10.1371/journal.ppat.1006313)

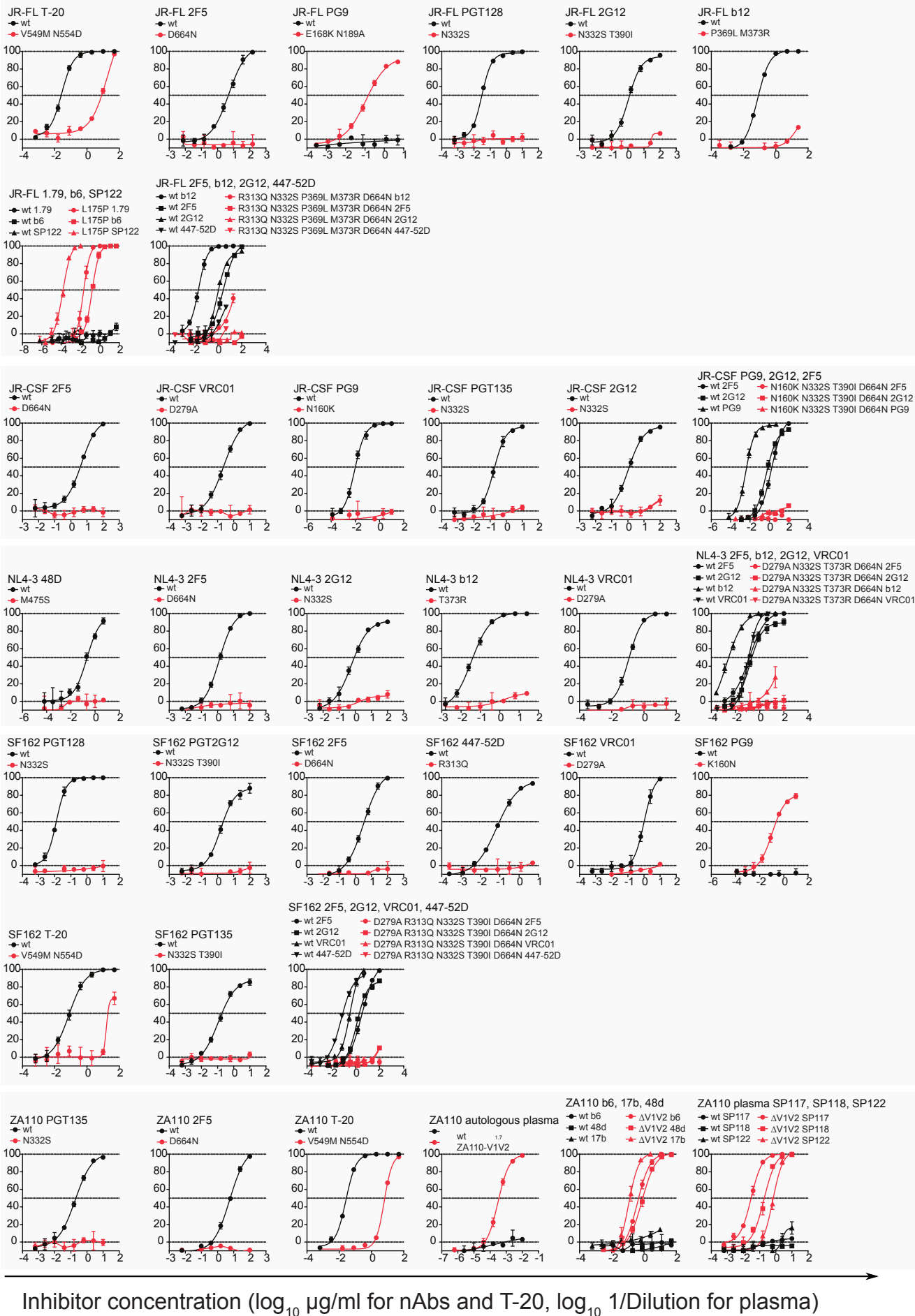

Supplement: S1 Fig — Our approach to estimate N relies on Env variants that are resistant or sensitive against neutralization by a given antibody (Fig 2A). Shown here are neutralization assays on TZM-bl cells with pseudotyped HIV-1 stocks carrying either wildtype (wt) or nAb escape or sensitivity mutant Envs as listed in S2 Table. Env variants and inhibitors tested are indicated above each panel. Only mutants showing complete escape from neutralization by the nAb of interest, or substantial loss in neutralization (<10% neutralization at a nAb concentration that results in 100% neutralization of the sensitive Env) were selected for further studies and are shown here. Data depict mean and SD from 2 independent experiments. (PDF) [file ppat.1006313.s001.pdf]

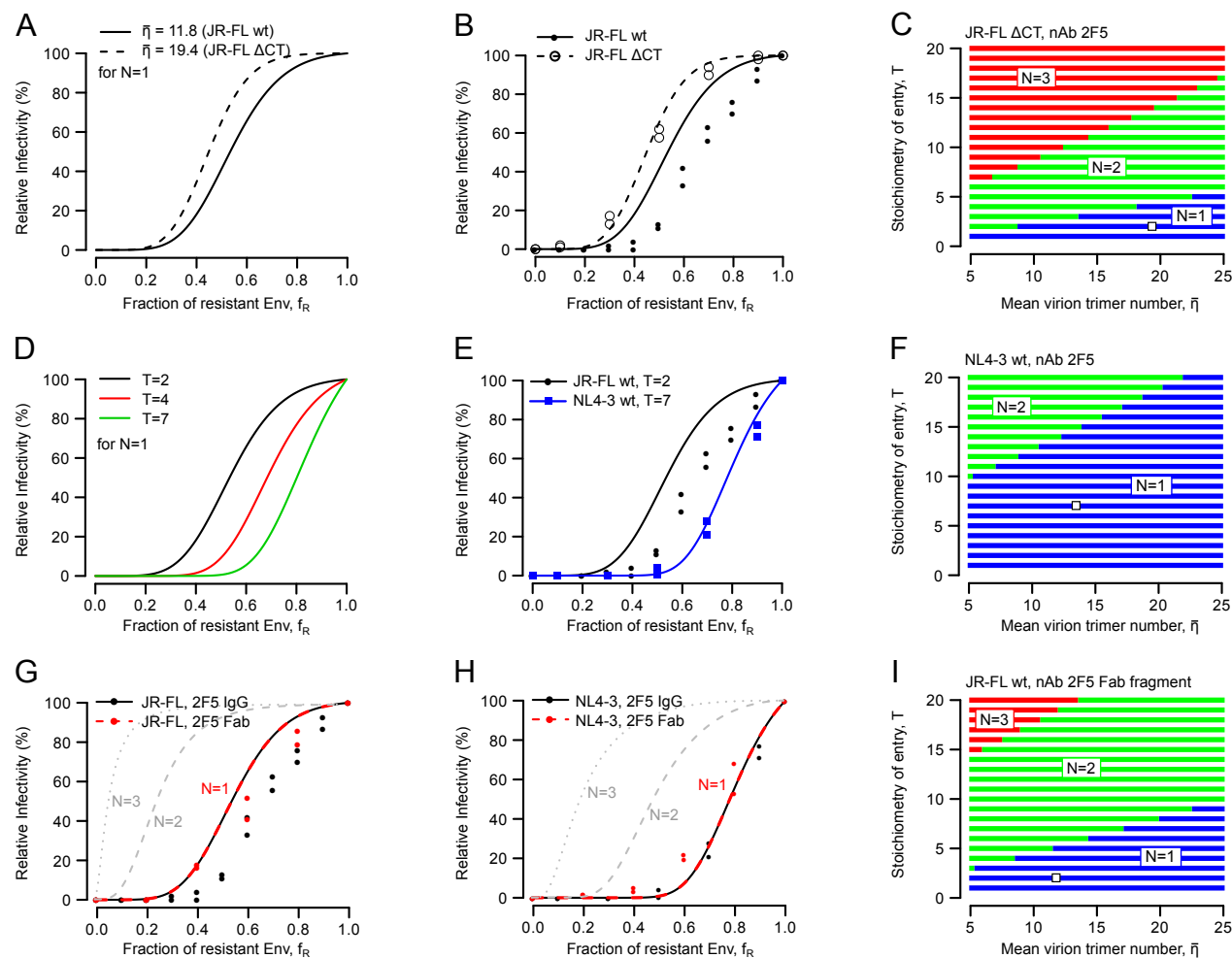

Supplement: S2 Fig — (A) Model predictions for the influence of η¯ on RI curve fits. (B) Mixed trimer assays with JR-FL wt (η¯=11.8) and JR-FL ΔCT (η¯=19.4) and nAb 2F5. The observed RI curve shifts are in agreement with shifts predicted by our model. (C) Data analysis revealed N = 1 for JR-FL ΔCT and nAb 2F5. (D) Model predictions for the influence of T on RI curve fits, assuming N = 1. (E) Mixed trimer assays with Envs JR-FL (T = 2) and NL4-3 (T = 7) and nAb 2F5. The observed RI curve shifts are in agreement with shifts predicted by our model. (F) Data analysis indicated N = 1 for NL4-3 and nAb 2F5. (G) and (H) To test the effect of mono versus bivalent nAb binding we compared 2F5 IgG and Fab fragment on strains JR-FL and NL4-3. Identical RI profiles for IgG and Fab fragments were observed. (I) Analysis of the JR-FL and NL4-3 2F5 Fab data indicated N = 1 in both cases. (PDF) [file ppat.1006313.s002.pdf]

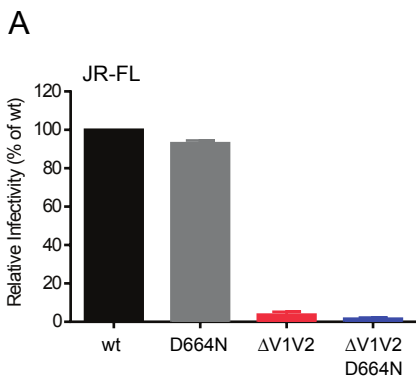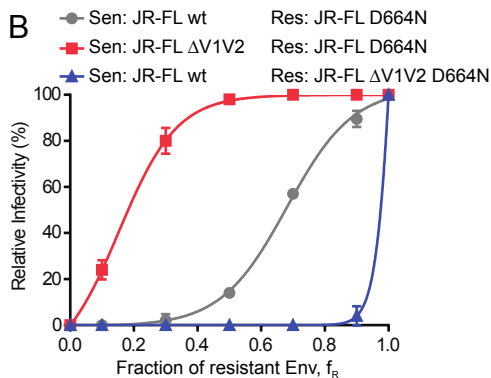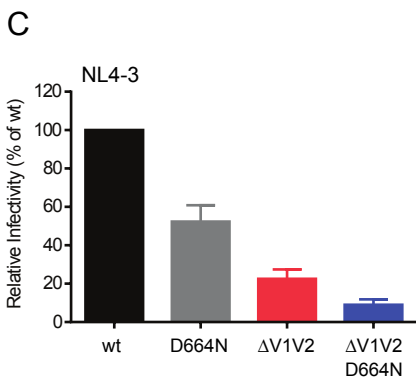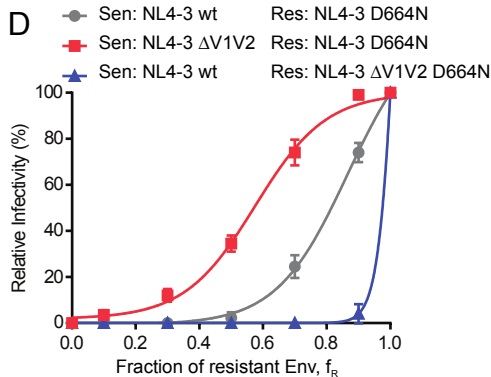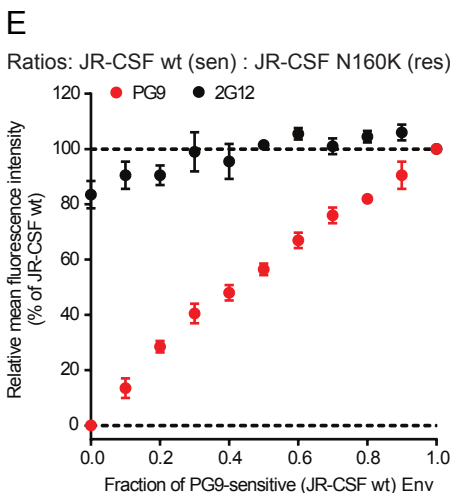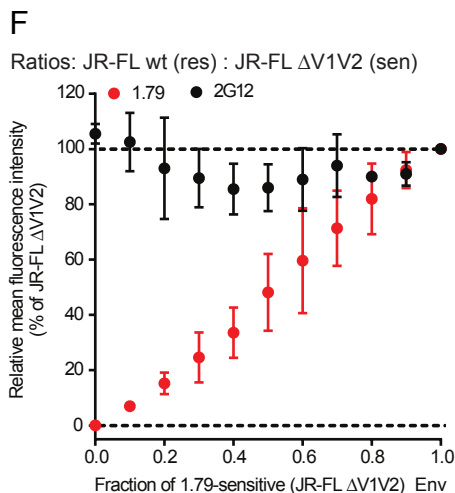

Supplement: S3 Fig — (A) Virus infectivity of JR-FL wt and indicated Env mutants, relative to JR-FL wt. D664N is a nAb 2F5 resistance mutation. (B) Ratio assays with nAb 2F5 and different combinations of JR-FL mixed trimer pseudovirus stocks. Combining JR-FL wt with JR-FL D664N, which have equal infectivity, yields a sigmoidal RI profile with an estimated N = 1 neutralization stoichiometry (Fig 2B and 2C). When the resistant Env (JR-FL D664N) is much more infectious than the sensitive Env (JR-FL ΔV1V2), the RI curve shifts strongly to the left (red squares). When the resistant Env (JR-FL ΔV1V2 D664N) is much less infectious than the sensitive Env (JR-FL wt), the RI curve shifts strongly to the right (blue triangles). We observed the same effects for HIV-1 strain NL4-3 (C and D). Thus, we only employed sensitive-resistant Env combinations with similar infectivities (within a two-fold range) for mathematical estimations of N. Interestingly, Env infectivity differences are not necessarily caused by expression differences (E and F). Expression of Envs on transfected 293-T cells was assessed by flow cytometry. (E) 293-T cells were transfected with the indicated ratios of JR-CSF wt and JR-CSF N160K, the latter being resistant to nAb PG9 and showing only 8% of JR-CSF wt infectivity (S2 Table). Both Envs are bound (and neutralized) equally well by nAb 2G12. The transfected 293-T cells were stained with both nAbs PG9 and 2G12 and analyzed by flow cytometry; the mean fluorescence intensity of each cell population in relation to cells expressing JR-CSF wt Env only are shown. Both Envs express to equal levels as judged by 2G12 staining. In addition, we observed a linear relation of nAb PG9 binding to cells in dependence on the ratio between PG9-sensitive and resistant Env, as expected. Thus, the low infectivity of JR-CSF N160K is not due to expression defects. (F) Identical analysis as in (E) for JR-FL wt and JR-FL ΔV1V2, the latter being highly neutralization sensitive and showing only 4% of JR-FL [file ppat.1006313.s003.pdf]

For all panels: — N=1 — N=2 — N=3

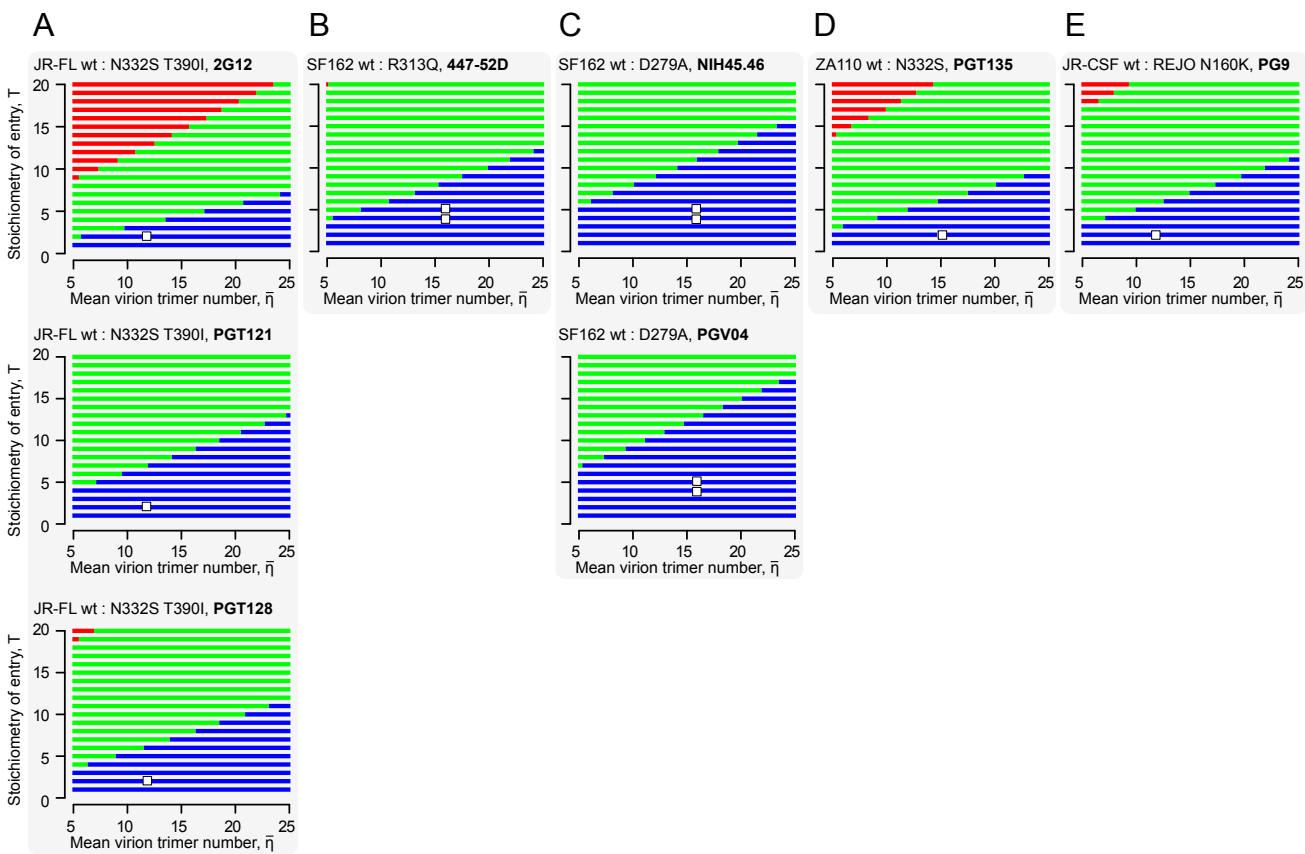

Supplement: S4 Fig — Shown are robustness analyses for the estimations of N shown in Fig 3A–3G. Each plot depicts the T and η¯ values assumed for the analysis of each individual Env combination (white dots; two dots are shown in case of divergent estimates of T for a given Env, see S3 Table). The resulting estimates of N in dependence of T and η¯ are color-coded: blue represents estimates of N = 1, green indicates estimates of N = 2, and red indicates estimates of N = 3. As shown, all N estimates are clearly within the N = 1 range. (PDF) [file ppat.1006313.s004.pdf]

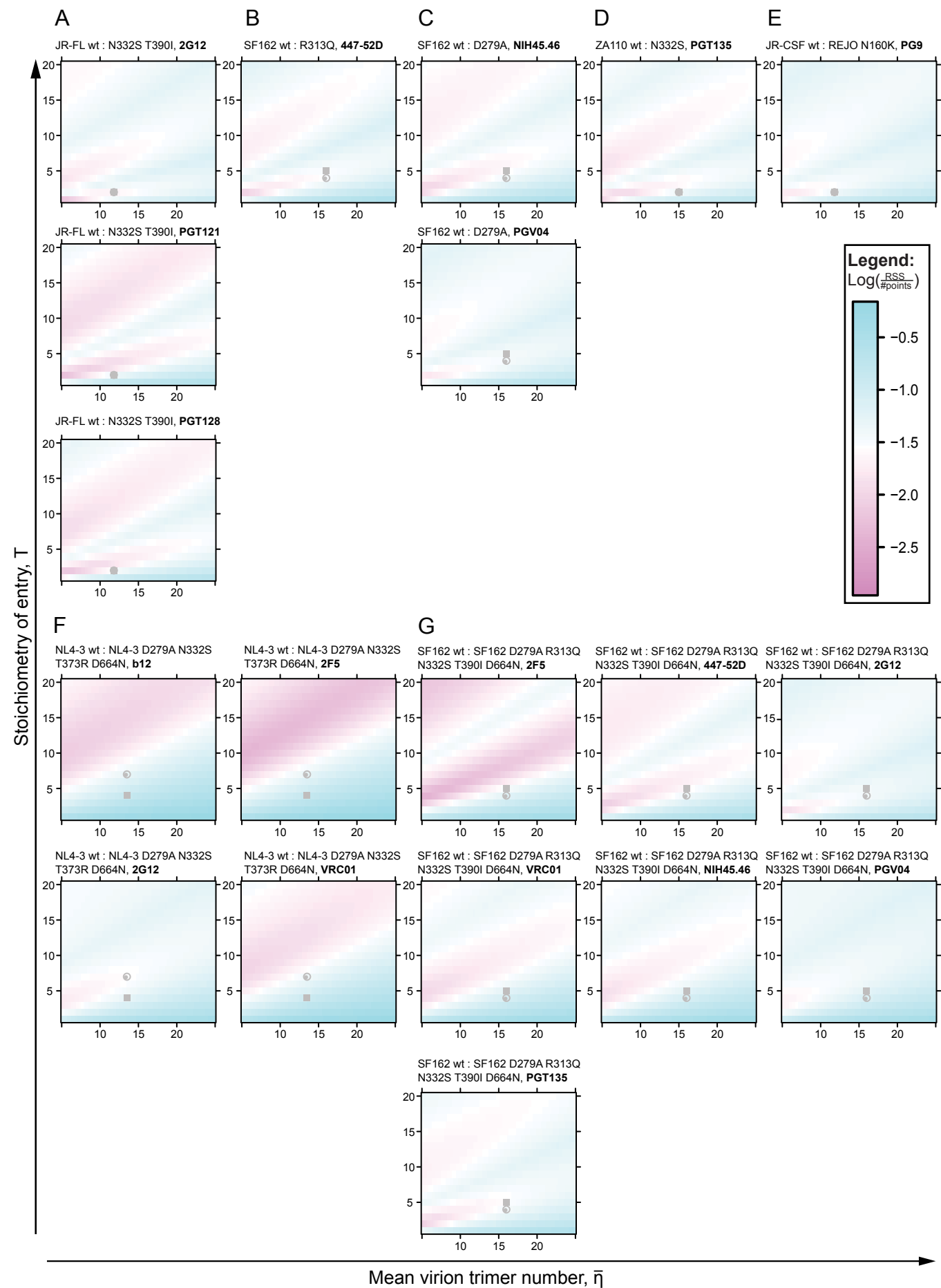

Supplement: S6 Fig — Goodness-of-fit analyses of all data shown in Fig 3. As shown in Fig 2E, better fits would, in most cases, be obtained for lower values of T and η¯. (PDF) [file ppat.1006313.s006.pdf]

A

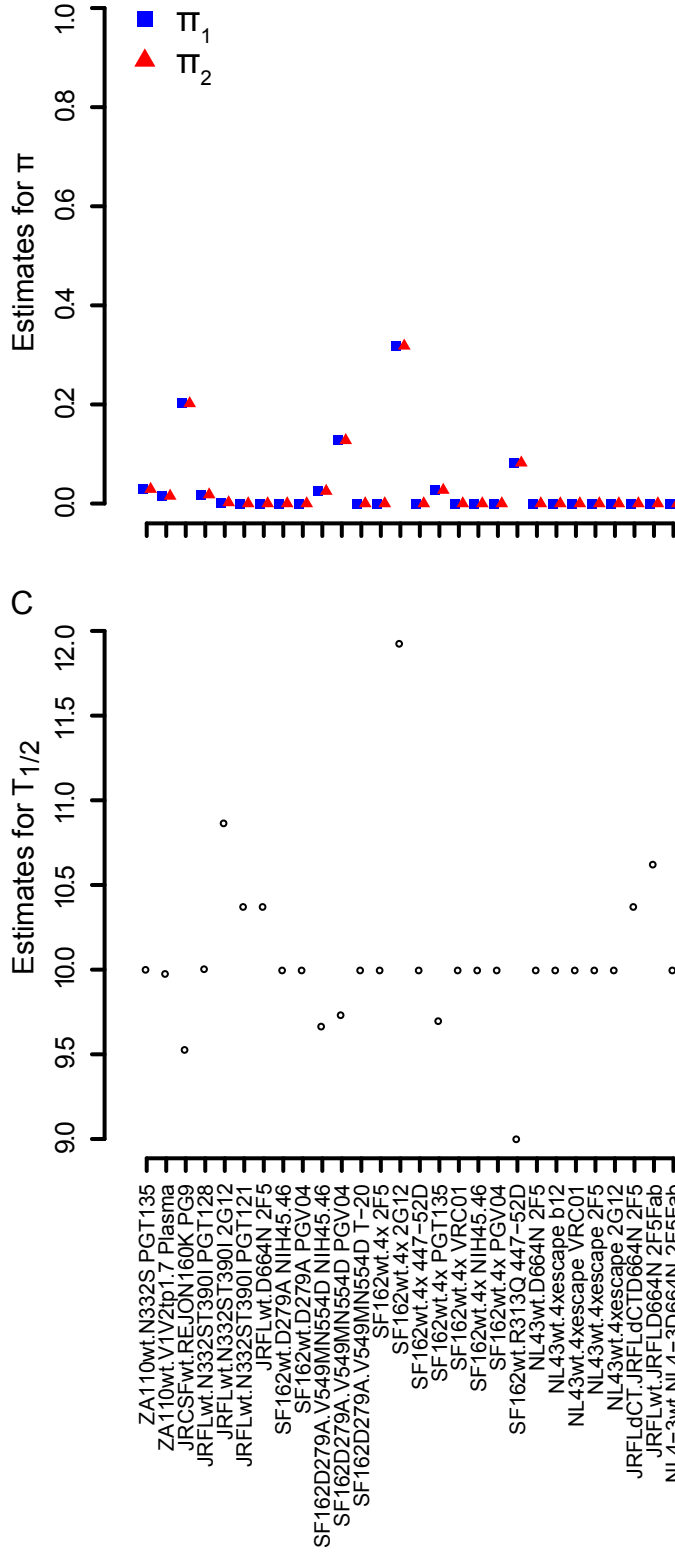

C

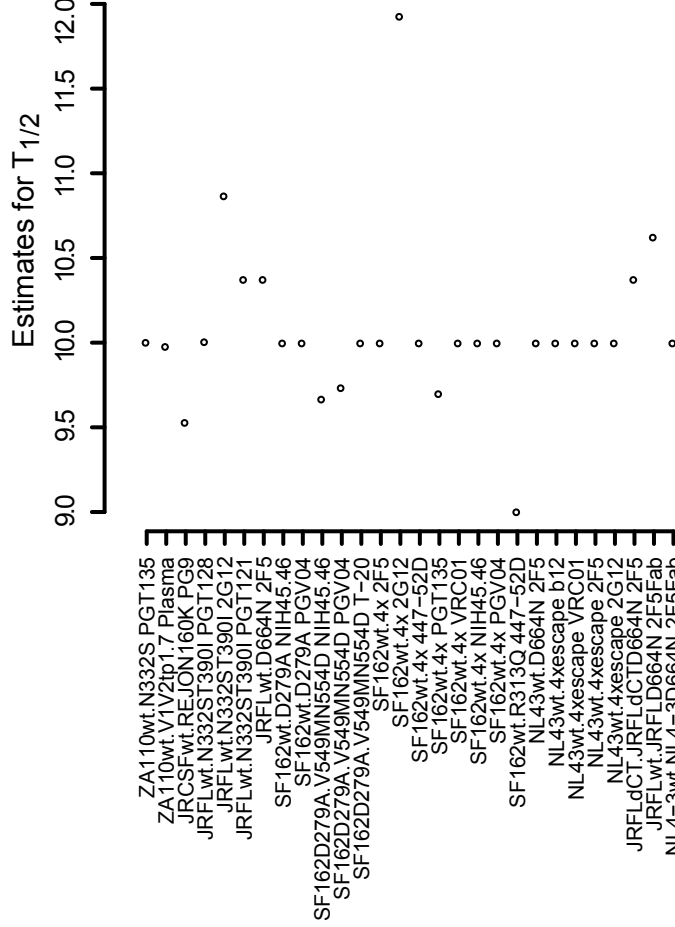

B

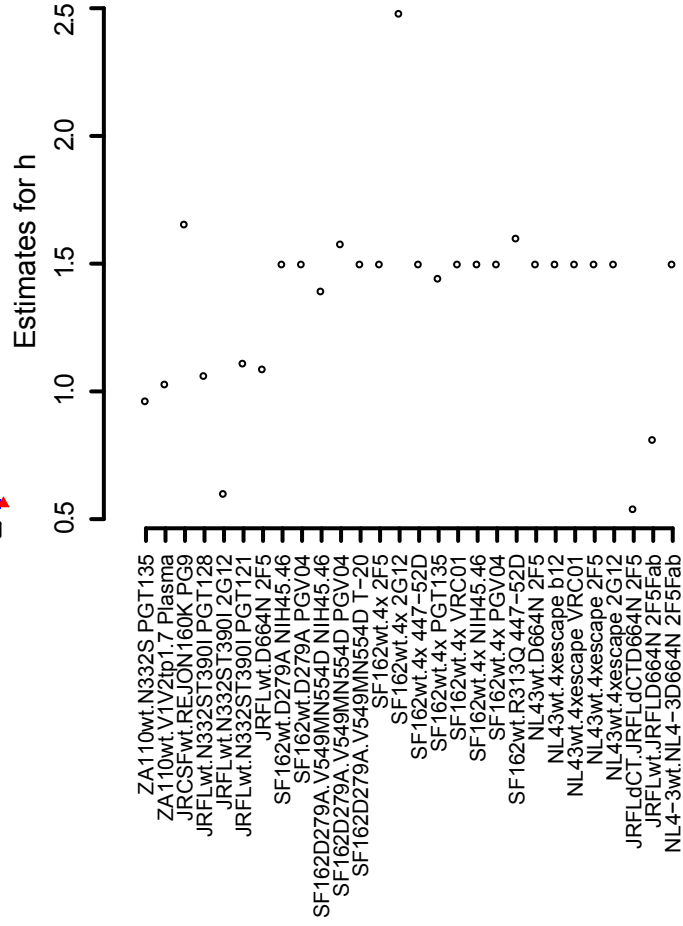

Supplement: S7 Fig — This model extension allows for partial trimer functionality loss upon antibody binding: the functionality of a trimer with one subunit bound to an antibody is π1, the functionality of a trimer with two bound subunits is π2, and a trimer with three subunits completely loses its functionality. In addition, the ability of a virion to infect a cell scales with the number of functional trimers, g, according to the equation gh/(gh +T1/2h). (A) Estimates of trimer functionality upon antibody binding. For most virus-nAb combinations, the trimer loses functionality upon binding of one antibody. (B) Estimates for the steepness of the infection curve, h, and (C) estimates for the half maximal trimer number, T1/2. A bootstrap procedure shows very high uncertainty in these point estimates. We therefore discarded this model extension in favor of a “hard” threshold model. (PDF) [file ppat.1006313.s007.pdf]

**A**

Env 1: SF162 D279A (res NIH45.46, PGV04)  
Env 2: SF162 V549M N554D (res T-20)

▲ NIH45.46  
◊ PGV04  
\* T-20

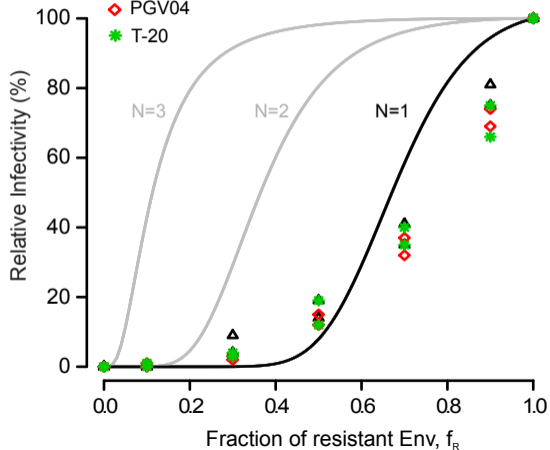**B**

Res: ZA110 V549M N554D  
Sen: ZA110  $\Delta$ V1V2

■ T-20  
● b6  
▲ 447-52D  
◆ PGT145

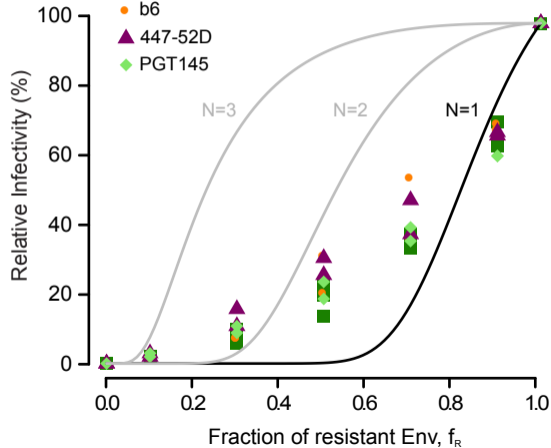

Supplement: S9 Fig — We utilized Env mixed trimer setups that allowed parallel assessment of both nAbs and the HIV-1 fusion inhibitor T-20 to obtain a direct comparison of the two inhibitor types. (A) Mixed trimer setup with Env mutants SF162 D279A and V549M N554D and nAbs NIH45.46, PGV04 and entry inhibitor T-20, indicating equal neutralization stoichiometry of N = 1. Note that in this setup the two Envs are reciprocally neutralization sensitive and resistant: SF162 D279A is resistant to nAbs PGV04 and NIH45.46 but sensitive to T-20; the opposite is true for SF162 V549M N554D. (B) Mixed trimer setup with Env mutants ZA110 ΔV1V2 and V549M N554D and nAbs b6, 447-52D and PGT145 and entry inhibitor T-20, indicating equal neutralization stoichiometry of N = 1. All graphs depict mean and SD from 2 independent experiments. (PDF) [file ppat.1006313.s009.pdf]

**A**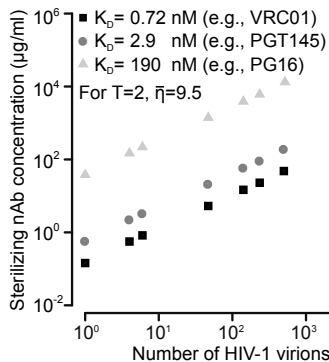**B**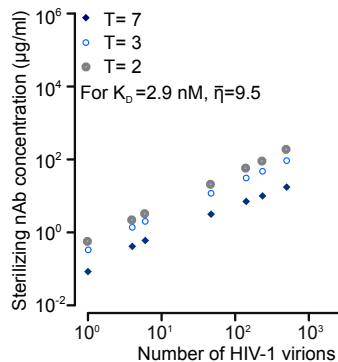**C**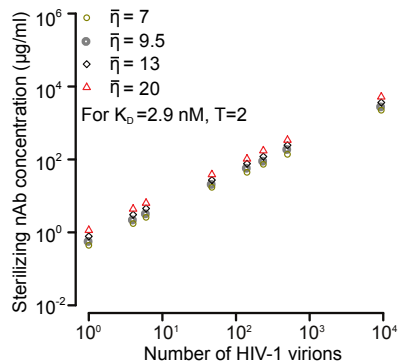**D**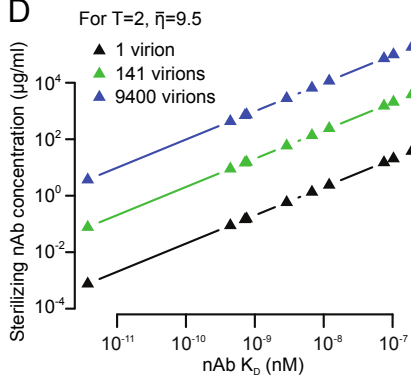**E**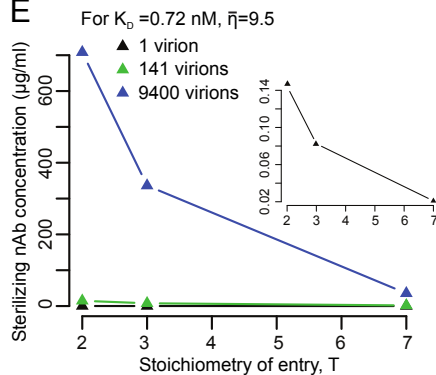**F**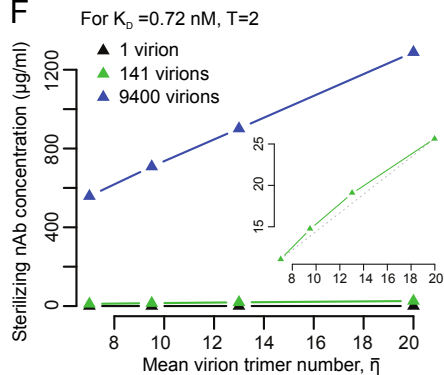

Supplement: S10 Fig — (A) to (C) Predicted nAb concentrations required to completely neutralize HIV-1 virion populations as a function of varying size depending on (A) nAb KD, (B) HIV-1 entry stoichiometry, T, and (C) mean viron trimer number, η¯. Panels (D) to (F) show the predicted nAb concentrations required to completely neutralize HIV-1 populations of 1, 141 or 9400 virions as a function of (D) nAb KD, (E) HIV-1 entry stoichiometry, T, or (F) the mean virion trimer number, η¯. The required nAb concentration for complete neutralization is a complex function of T and η¯, but linear in KD. This is the case because for a fixed viron population size, the fraction of envelope subunits required to be bound by nAb for complete neutralization, fb, is constant (see also Fig 4F). (PDF) [file ppat.1006313.s010.pdf]

A

Virion trimer number distributions:

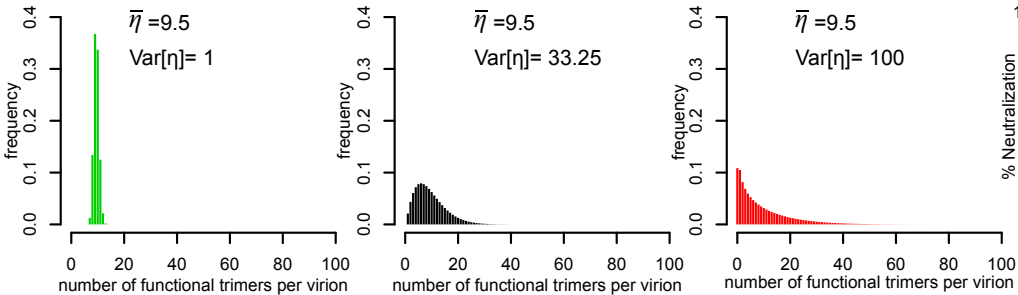

B

Predicted neutralization curves:

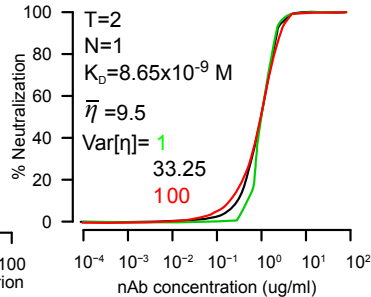

Supplement: S12 Fig — To follow up the discrepancy between experimental and predicted neutralization curve steepness (i.e., Hill coefficient; see S11A Fig) we asked which parameter of our model may steer the steepness of the predicted curves. We found that assuming a broader virion trimer number distribution, i.e. a higher variance in trimer numbers across virions, results in less steep predicted neutralization curves. For this graph we used the following parameters: η¯=9.5, T = 2, N = 1, KD = 8.65x10^-9^. Green: Var[η] = 1, black: Var[η] = 33.25, red: Var[η] = 100. (PDF) [file ppat.1006313.s012.pdf]

A

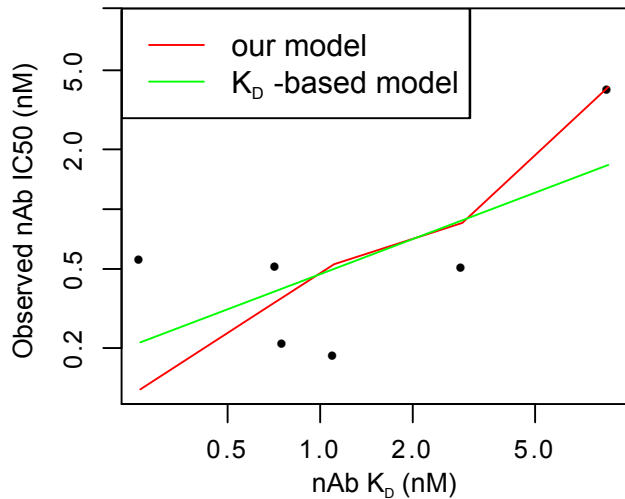

B

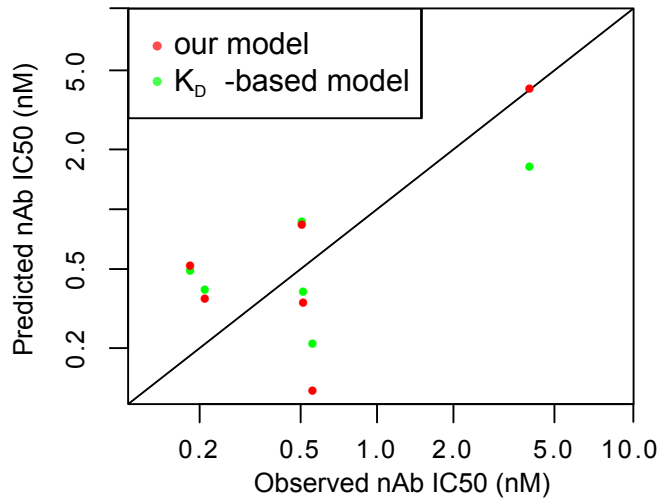

Supplement: S13 Fig — We defined the nAb KD-based model as a linear model between the logarithm of the observed nAb IC50s and the logarithm of the nAb KDs. (A) Our model (red line, shown for T = 3) follows fluctuations in the mean nAb IC50s better (black dots, for the nAb data see S5 Table) than the KD-based model (green line). (B) Predicted nAb IC50 values plotted against the mean of the observed IC50 values are shown in green for the KD-based model and in red for our model. The visually better performance in predicting IC50 values of our model in comparison to a KD-based model is statistically confirmed by using the Akaike Information Criterion (AIC). We obtain lower AIC values for our model (AIC = 9.72 for our model with T = 2 and AIC = 8.7 for our model with T = 3) compared to an AIC = 11.7 for the KD-based model. Note that the value for 2G12 (around 5 nM KD) is not an outlier, as a Kolmogorov-Smirnov test for normality shows that the IC50 values are not distributed different from normal (p-value = 0.16). (PDF) [file ppat.1006313.s013.pdf]

Virion probability to start host infection,  $\psi$

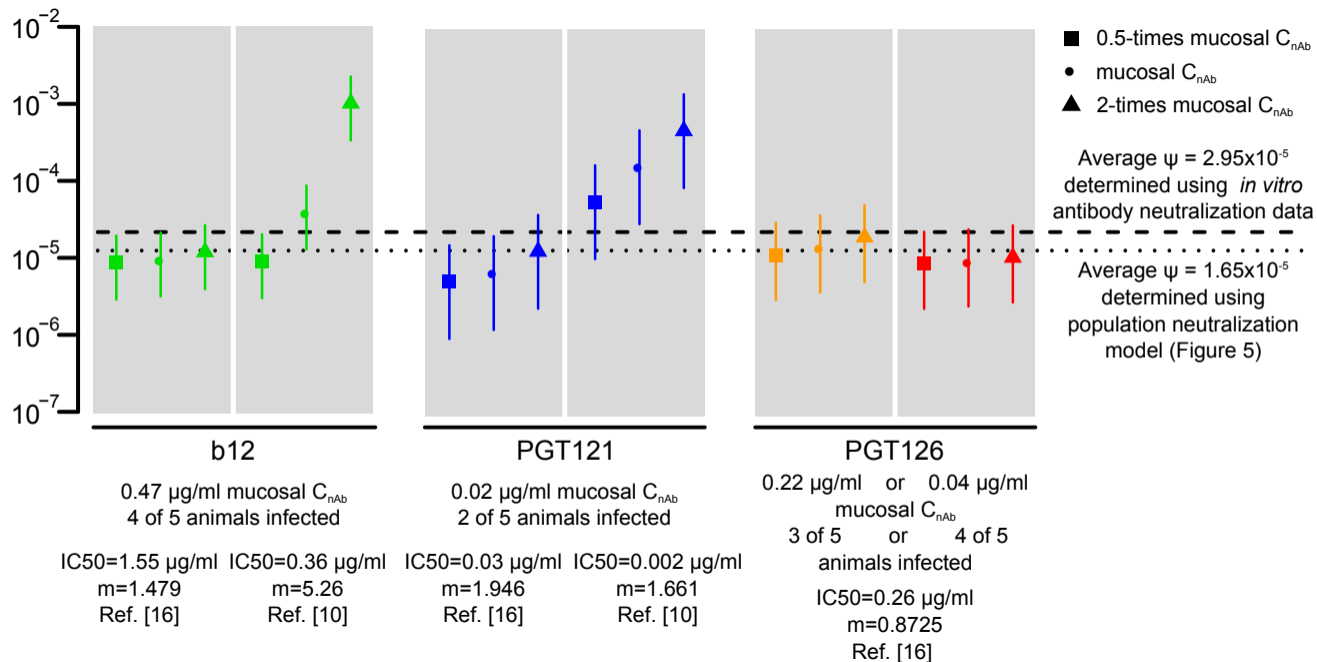

Supplement: S15 Fig — Having obtained similar ψ estimates across the four analyzed macaque challenge studies using our mechanistic model (Fig 5), we asked whether similar results could be obtained using solely in vitro nAb neutralization data (thus bypassing the need to have estimates for T, η¯ and nAb KD). We adjusted our model accordingly, requiring solely nAb IC50 and Hill coefficient as input. In vitro neutralization data of SHIV strain P3 with nAbs PGT121, PGT126 and b12 were previously reported [10, 16] and are shown in S11 Fig. Utilizing these data we obtained a closely matching ψ value of 2.95x10^-5^. We thus conclude that (i) the estimate of ψ is likely robust within the range of the two estimated values (i.e., between 1.65 and 2.95 x10^-5^), and (ii) in cases where information on T, η¯ and nAb KD are missing, in vitro nAb neutralization data may provide a good substitute to analyze macaque challenge studies as proposed herein. (PDF) [file ppat.1006313.s015.pdf]

**A**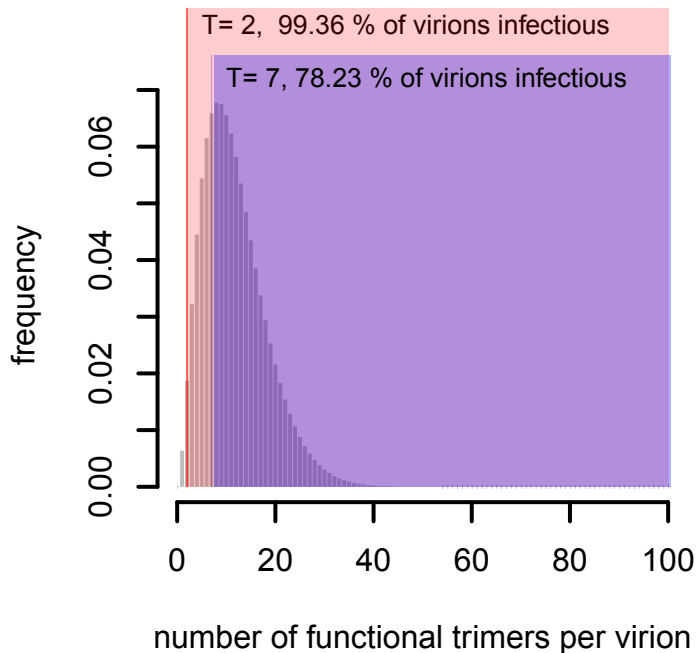**B**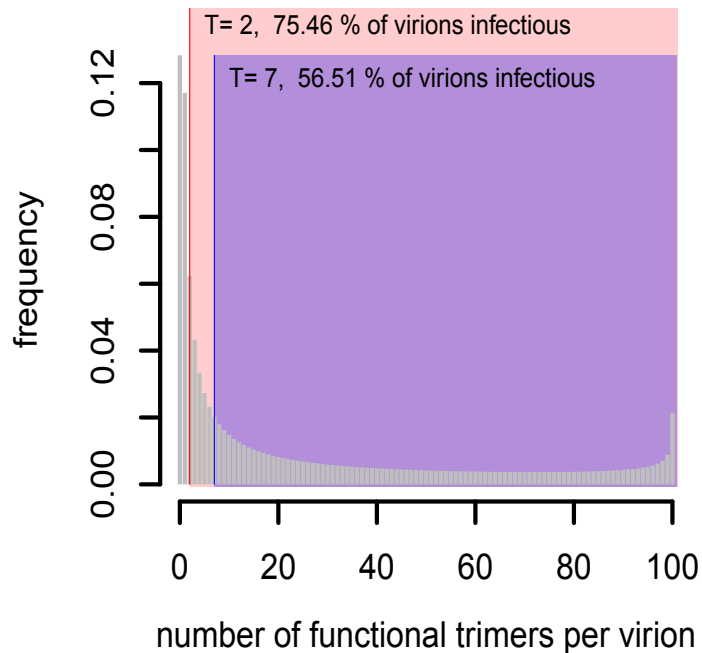

Supplement: S16 Fig — Shown are two hypothetical virion trimer number distributions, and how they influence the fraction of infectious virions in the population in dependence on T. (A) η¯=11.8, Var[η] = 41.3. (B) η¯=11.8, Var[η] = 600. (PDF) [file ppat.1006313.s016.pdf]
