# Supplementary material for: Predicting HIV-1 transmission and antibody neutralization efficacy in vivo from stoichiometric parameters
Source: PLoS Pathog. 2017 May 4;13(5):e1006313. doi: 10.1371/journal.ppat.1006313 (PMC5417720; doi:10.1371/journal.ppat.1006313)
Supplement: S5 Fig — Bootstrap analysis with 1000 replicates of all data shown in Fig 3, indicating that the N = 1 estimate is accurate. (PDF) [file ppat.1006313.s005.pdf]

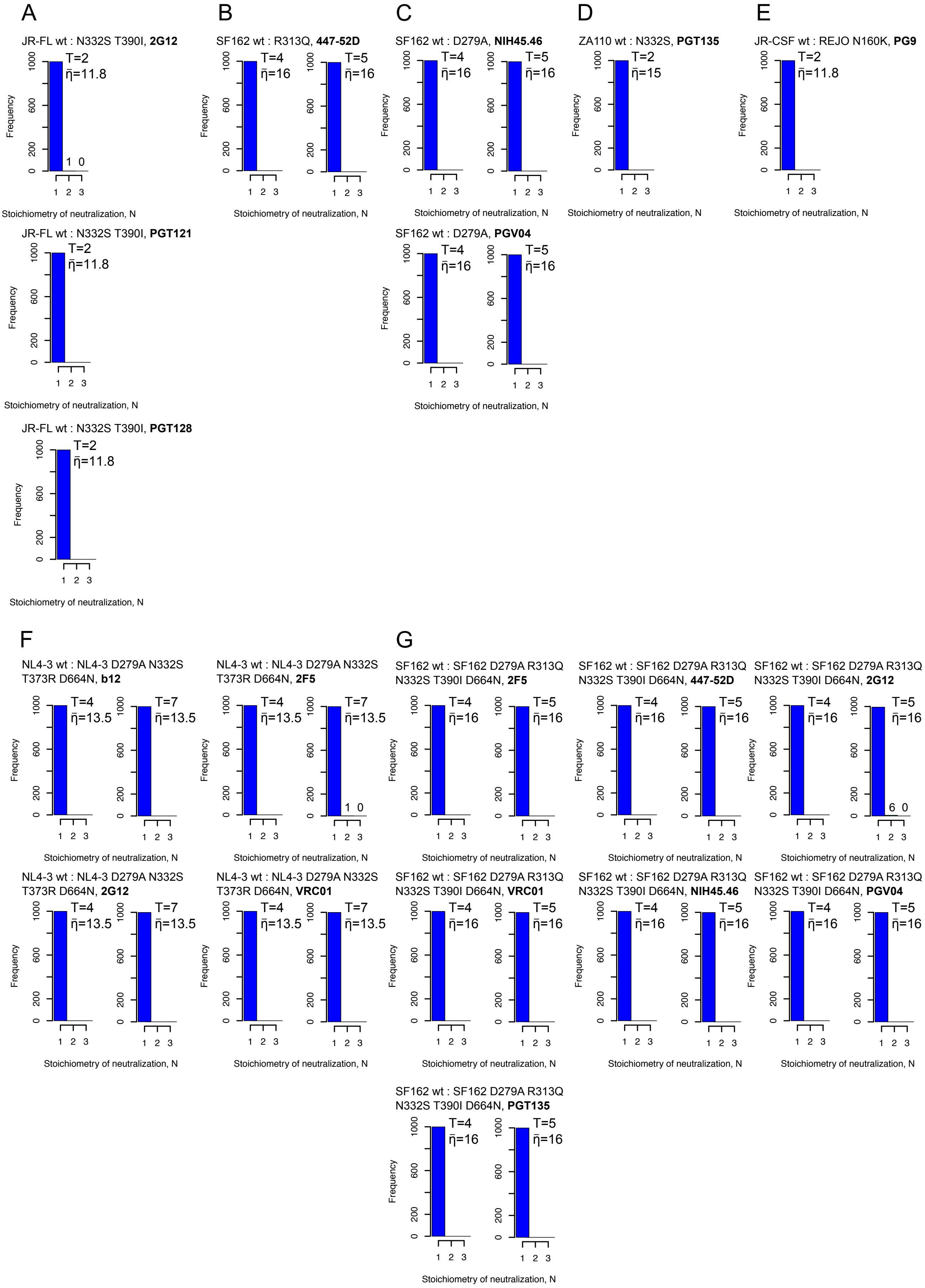
A
JR-FL wt : N332S T390I, **2G12**
T=2
η̄=11.8
1 0
Frequency
Stoichiometry of neutralization, N
JR-FL wt : N332S T390I, **PGT121**
T=2
η̄=11.8
JR-FL wt : N332S T390I, **PGT128**
T=2
η̄=11.8
B
SF162 wt : R313Q, **447-52D**
T=4
η̄=16
T=5
η̄=16
C
SF162 wt : D279A, **NIH45.46**
T=4
η̄=16
T=5
η̄=16
SF162 wt : D279A, **PGV04**
T=4
η̄=16
T=5
η̄=16
D
ZA110 wt : N332S, **PGT135**
T=2
η̄=15
E
JR-CSF wt : REJO N160K, **PG9**
T=2
η̄=11.8
F
NL4-3 wt : NL4-3 D279A N332S T373R D664N, **b12**
T=4
η̄=13.5
T=7
η̄=13.5
NL4-3 wt : NL4-3 D279A N332S T373R D664N, **2F5**
T=4
η̄=13.5
T=7
η̄=13.5
1 0
NL4-3 wt : NL4-3 D279A N332S T373R D664N, **2G12**
T=4
η̄=13.5
T=7
η̄=13.5
NL4-3 wt : NL4-3 D279A N332S T373R D664N, **VRC01**
T=4
η̄=13.5
T=7
η̄=13.5
G
SF162 wt : SF162 D279A R313Q N332S T390I D664N, **2F5**
T=4
η̄=16
T=5
η̄=16
SF162 wt : SF162 D279A R313Q N332S T390I D664N, **447-52D**
T=4
η̄=16
T=5
η̄=16
SF162 wt : SF162 D279A R313Q N332S T390I D664N, **2G12**
T=4
η̄=16
T=5
η̄=16
6 0
SF162 wt : SF162 D279A R313Q N332S T390I D664N, **VRC01**
T=4
η̄=16
T=5
η̄=16
SF162 wt : SF162 D279A R313Q N332S T390I D664N, **NIH45.46**
T=4
η̄=16
T=5
η̄=16
SF162 wt : SF162 D279A R313Q N332S T390I D664N, **PGV04**
T=4
η̄=16
T=5
η̄=16
SF162 wt : SF162 D279A R313Q N332S T390I D664N, **PGT135**
T=4
η̄=16
T=5
η̄=16
