# Supplementary material for: Predicting HIV-1 transmission and antibody neutralization efficacy in vivo from stoichiometric parameters
Source: PLoS Pathog. 2017 May 4;13(5):e1006313. doi: 10.1371/journal.ppat.1006313 (PMC5417720; doi:10.1371/journal.ppat.1006313)
Supplement: S8 Fig — (A) to (C): Envs with multiple nAb resistance mutations allowed parallel assessment of various nAbs on the same set of mixed trimer virus stocks. The employed resistant and sensitive Envs and nAbs tested are indicated above each panel. The three mixed trimer setups shown here were not infectivity-matched, excluding mathematical estimation of N. However, inclusion of nAb 2F5, previously shown to neutralize with N = 1 (Fig 2), and graphic comparison of all RI curves indicates equal N (N = 1) of all nAbs tested. (PDF) [file ppat.1006313.s008.pdf]

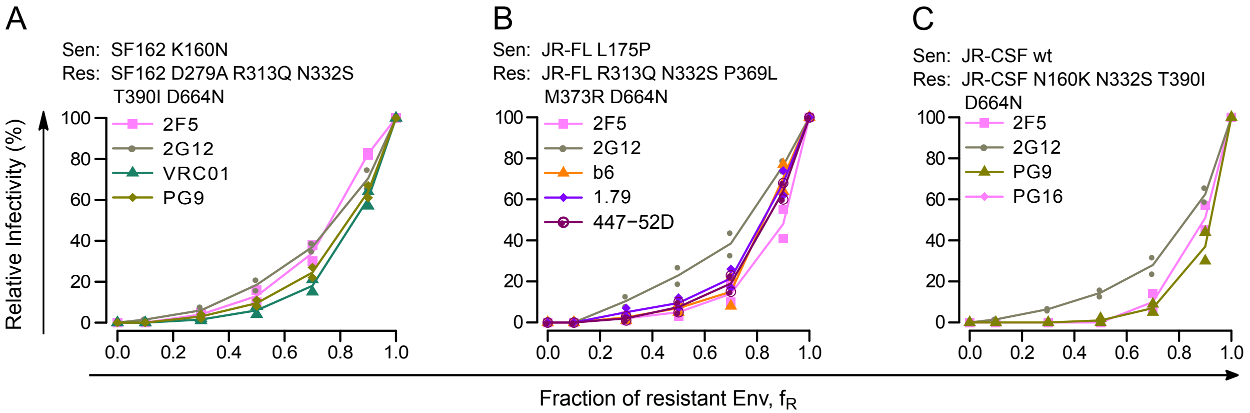
A
Sen: SF162 K160N
Res: SF162 D279A R313Q N332S T390I D664N
2F5
2G12
VRC01
PG9
B
Sen: JR-FL L175P
Res: JR-FL R313Q N332S P369L M373R D664N
2F5
2G12
b6
1.79
447−52D
C
Sen: JR-CSF wt
Res: JR-CSF N160K N332S T390I D664N
2F5
2G12
PG9
PG16
Relative Infectivity (%)
100
80
60
40
20
0
0.0
0.2
0.4
0.6
0.8
1.0
Fraction of resistant Env, f_R
