# Supplementary material for: Predicting HIV-1 transmission and antibody neutralization efficacy in vivo from stoichiometric parameters
Source: PLoS Pathog. 2017 May 4;13(5):e1006313. doi: 10.1371/journal.ppat.1006313 (PMC5417720; doi:10.1371/journal.ppat.1006313)
Supplement: S11 Fig — (A) Comparison of predicted and experimental nAb neutralization curves for HIV-1 strain BG505 across five nAbs. BG505-specific T, η¯ and nAb KD values (see S3 and S5 Tables) were used to predict the neutralization curves. Experimental data were obtained using BG505 pseudovirus stocks and TZM-bL reporter cells. We find that the predicted neutralization curves are notably steeper than experimentally obtained curves. (B) Comparison of predicted and experimental nAb neutralization curves for SHIV strain P3 across three nAbs. P3-specific T, η¯ and nAb KD values (see S3 Table) were used to predict the neutralization curves. Experimental data were obtained using replication-competent SHIV-P3 stocks and PBMC target cells, and were previously reported [10, 16]. We noted that especially for nAbs PGT121 and b12, predicted and experimental neutralization curves showed better agreement than for HIV-1 BG505 in (A). (PDF) [file ppat.1006313.s011.pdf]

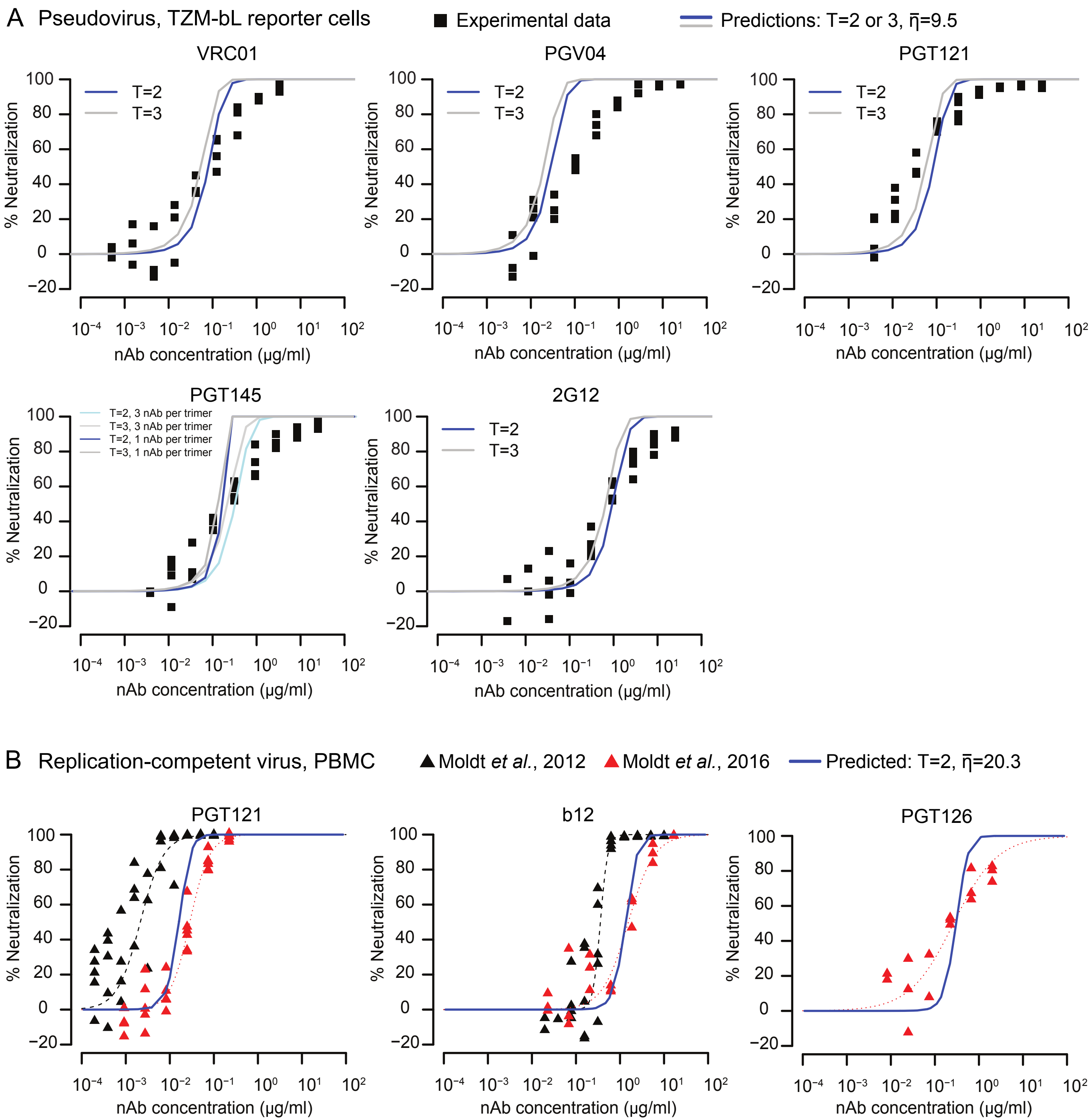
A Pseudovirus, TZM-bL reporter cells
Experimental data
Predictions: T=2 or 3, η̄=9.5
VRC01
PGV04
PGT121
PGT145
2G12
T=2
T=3
T=2, 3 nAb per trimer
T=3, 3 nAb per trimer
T=2, 1 nAb per trimer
T=3, 1 nAb per trimer
% Neutralization
nAb concentration (μg/ml)
B Replication-competent virus, PBMC
Moldt et al., 2012
Moldt et al., 2016
Predicted: T=2, η̄=20.3
PGT121
b12
PGT126
