# Supplementary material for: Predicting HIV-1 transmission and antibody neutralization efficacy in vivo from stoichiometric parameters
Source: PLoS Pathog. 2017 May 4;13(5):e1006313. doi: 10.1371/journal.ppat.1006313 (PMC5417720; doi:10.1371/journal.ppat.1006313)
Supplement: S14 Fig — (A) Predicted neutralization curves for nAbs b12, 2G12, PGT121 and PGT126 against the challenge virus SHIV-P3. Shaded areas indicate 2-fold variation in nAb KD. Dots depict measured or extrapolated vaginal nAb concentrations and the coloured areas indicate 2-fold variation in these nAb concentrations, indicating the SHIV-P3 predicted neutralization ranges achieved in the three studies. (B) The fraction of predicted non-neutralized SHIV-P3 virions in dependence on mucosal nAb concentrations, as shown in (A). (PDF) [file ppat.1006313.s014.pdf]

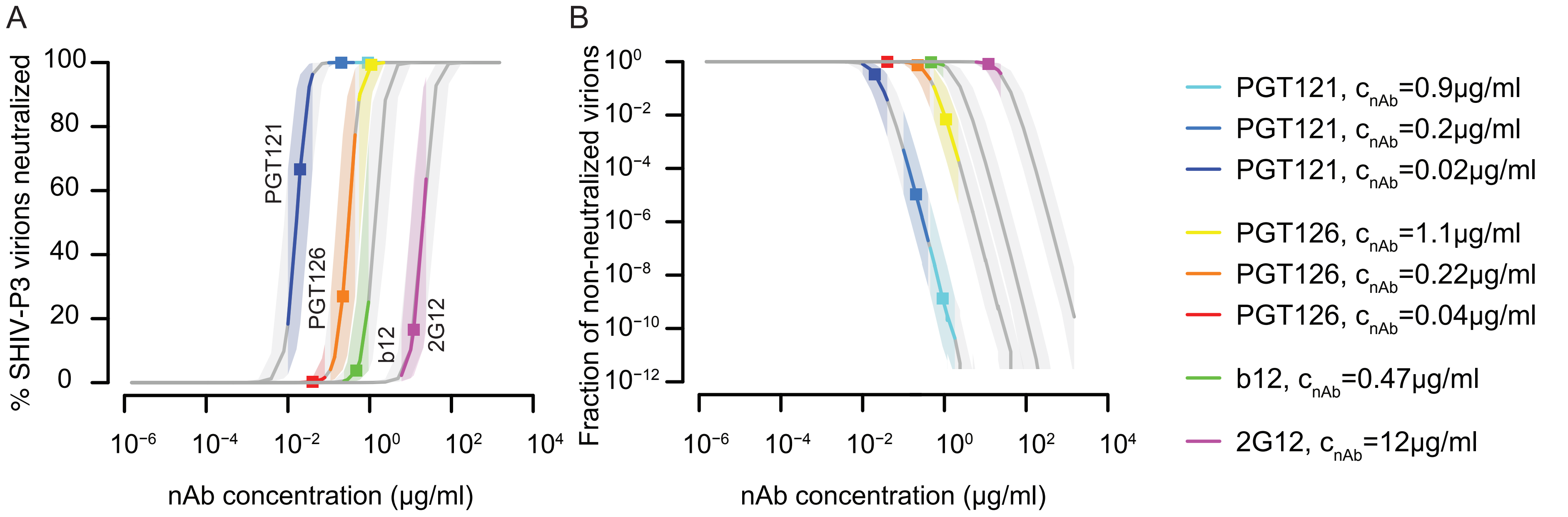
A
% SHIV-P3 virions neutralized
100
80
60
40
20
0
PGT121
PGT126
b12
2G12
10^−6
10^−4
10^−2
10^0
10^2
10^4
nAb concentration (µg/ml)
B
Fraction of non-neutralized virions
10^0
10^−2
10^−4
10^−6
10^−8
10^−10
10^−12
10^−6
10^−4
10^−2
10^0
10^2
10^4
nAb concentration (µg/ml)
PGT121, c_nAb=0.9µg/ml
PGT121, c_nAb=0.2µg/ml
PGT121, c_nAb=0.02µg/ml
PGT126, c_nAb=1.1µg/ml
PGT126, c_nAb=0.22µg/ml
PGT126, c_nAb=0.04µg/ml
b12, c_nAb=0.47µg/ml
2G12, c_nAb=12µg/ml
