# Supplementary material for: Predicting HIV-1 transmission and antibody neutralization efficacy in vivo from stoichiometric parameters
Source: PLoS Pathog. 2017 May 4;13(5):e1006313. doi: 10.1371/journal.ppat.1006313 (PMC5417720; doi:10.1371/journal.ppat.1006313)
Supplement: S1 Table — (DOCX) [file ppat.1006313.s017.docx]

**S1 Table: Parameters required for modelling HIV-1 virion population neutralization by antibodies**

| **Parameter** | **Description** | **Value** | **References** |
| --- | --- | --- | --- |
| **N** | Stoichiometry of neutralization | N = 1 | Estimated here |
| **T** | Stoichiometry of entry | T = 2 to 7  Depending on viral strain | Ref [1] |
| **η** | Virion trimer number distribution | Discretized B-distribution with mean $\bar{\eta}$ and variance Var(η) | Defined in Ref [2] |
| $\bar{\eta}$ | Mean virion trimer number | 6.7 to 20.3 trimers per virion, depending on viral strain | Ref [1] |
| **Var(η)** | Variance of virion trimer numbers | 49/14 $\bar{\eta}$ | Ref [3] |
| **K_D_** | Antibody trimer binding constant | Depending on the antibody and the viral envelope | For BG505:  Ref [4] |
| **IC50** | Antibody concentration at which 50% inhibition is reached | Depending on the antibody and the viral envelope. IC50s are given in µg/ml. As the molar mass of an antibody is 150 kg/mol, one can calculate the molar IC50 with [(IC50 in µg/ml)/150]*10^-6^M | Various sources |
| **c_Ab_** | Antibody concentration | Variable | N.A. |
| **c_sem_(vir)** | Virus concentration in semen | Typically 10^2^ to 10^4^ RNA copies per mL semen during chronic infection | Ref [5] |
| **v_sem_** | Semen volume | Typically between 1 and 10 mL | Ref [6] |
| **n_in_** | HIV-1 inoculum size in penile-vaginal transmission | C_sem_(vir) * v_sem_  Modelled from 10 to 10^7^ virions | N.A. |
| **p_pen_** | Probability that a virion penetrates the vaginal mucosal epithelium | 0.00235 | Derived from Ref [7] |
| **p_inf_** | Probability that an animal becomes infected in a challenge study; depends on viral inoculum, antibody concentration and other experimental factors | Maximum-likelihood estimation from challenge studies.  Geometric distribution  or binomial distribution, depending on the challenge regime. | Estimated here based on data from:  Refs [8-11] |
| **ψ** | Probability of an infectious virion to start a host infection | 0.0000165 | Estimated here |
