# Supplementary material for: Predicting HIV-1 transmission and antibody neutralization efficacy in vivo from stoichiometric parameters
Source: PLoS Pathog. 2017 May 4;13(5):e1006313. doi: 10.1371/journal.ppat.1006313 (PMC5417720; doi:10.1371/journal.ppat.1006313)
Supplement: S2 Table — (DOCX) [file ppat.1006313.s018.docx]

**S2 Table: Env antibody escape or sensitivity mutants**

| **Strain** | **Variant** | **Neutralization phenotype** | **Infectivity (% of wt)** |
| --- | --- | --- | --- |
| **JR-FL** | **wt** | **moderately neutralization resistant (tier 2)** | **100** |
| JR-FL | L175P | highly neutralization sensitive | 25.3 |
| JR-FL | N332S | PGT128, PGT121 resistant | 82.8 |
| JR-FL | D664N | 2F5 resistant | 93.0 |
| JR-FL | E168K N189A | PG9 sensitive | 62.7 |
| JR-FL | N332S T390I | PGT128, PGT121, 2G12 resistant | 85.7 |
| JR-FL | P369L M373R | b12 resistant | 32.3 |
| JR-FL | V549M N554D | T-20 resistant | 9.3 |
| JR-FL | P369L M373R D664N | b12, 2F5 resistant | 31.0 |
| JR-FL | N332S P369L M373R D664N | b12, 2F5, PGT128, PGT121, 2G12 resistant | 18.5 |
| JR-FL | R313Q N332S P369L M373R D664N | b12, 2F5, PGT128, PGT121, 2G12, 447-52D resistant | 6.0 |
| JR-FL | ΔV1V2 | highly neutralization sensitive | 3.8 |
| JR-FL | ΔV1V2 D664N | highly neutralization sensitive, 2F5 resistant | 1.7 |
| **JR-CSF** | **wt** | **moderately neutralization resistant (tier 2)** | **100** |
| JR-CSF | N160K | PG9 resistant | 8.3 |
| JR-CSF | D279A | VRC01, NIH45.46, PGV04 resistant | 11.8 |
| JR-CSF | N332S | PGT135 resistant | 90.7 |
| JR-CSF | D664N | 2F5 resistant | 106.7 |
| JR-CSF | N160K N332S T390I D664N | PG9, 2G12, 2F5 resistant | 10.0 |
| **ZA110** | **wt** | **moderately neutralization resistant (tier 2)** | **100** |
| ZA110 | N332S | PGT135 resistant | 110.7 |
| ZA110 | D664N | 2F5 resistant | 38.0 |
| ZA110 | V549M N554D | T-20 resistant | 34.7 |
| ZA110 | ΔV1V2 | highly neutralization sensitive | 27.7 |
| ZA110 | V1V2 timepoint 1.7 | sensitive against autologous plasma | 74.7 |
| **S2 Table, continued** | | | |
| **Strain** | **Variant** | **Neutralization phenotype** | **Infectivity (% of wt)** |
| **SF162** | **wt** | **neutralization sensitive (tier 1)** | **100** |
| SF162 | K160N | PG9 sensitive | 325.8 |
| SF162 | D279A | VRC01, NIH45.46, PGV04 resistant | 77.3 |
| SF162 | R313Q | 447-52D resistant | 197.3 |
| SF162 | N332S | PGT135 resistant | 80.5 |
| SF162 | D664N | 2F5 resistant | 96.7 |
| SF162 | N332S T390I | PGT135, 2G12 resistant | 84.3 |
| SF162 | V549M N554D | T-20 resistant | 51.7 |
| SF162 | D279A R313Q N332S T390I D664N | VRC01, NIH45.46, PGV04, 447-52D, PGT135, 2G12, 2F5 resistant | 72.3 |
| **NL4-3** | **wt** | **neutralization sensitive (tier 1)** | **100** |
| NL4-3 | D279A | VRC01, NIH45.46, PGV04 resistant | 81.0 |
| NL4-3 | T373R | b12 resistant | 27.3 |
| NL4-3 | M475S | 17b, 48d resistant | 92.7 |
| NL4-3 | D664N | 2F5 resistant | 52.3 |
| NL4-3 | N332S T390I | 2G12 resistant | 23.3 |
| NL4-3 | D279A N332S T373R D664N | VRC01, 2G12, b12, 2F5 resistant | 54.0 |
| NL4-3 | ΔV1V2 | highly neutralization sensitive | 22.5 |

To compare virus infectivity across the variants of a given strain, all variant virus stocks were produced and titrated in parallel to avoid experimental variation. Infectivity values are derived from two independently produced and titrated batches of virus stocks and given as the average of these experiments; the infectivity of the corresponding wt variant is always set to 100%.
