# Supplementary material for: Predicting HIV-1 transmission and antibody neutralization efficacy in vivo from stoichiometric parameters
Source: PLoS Pathog. 2017 May 4;13(5):e1006313. doi: 10.1371/journal.ppat.1006313 (PMC5417720; doi:10.1371/journal.ppat.1006313)
Supplement: S3 Table — (DOCX) [file ppat.1006313.s019.docx]

**S3 Table: Entry parameters and origins of HIV-1 strains**

| **Strain** | **Variant** | **T (R508S/R511S)^a^** | **T (V513E)^a^** | **Virion trimer number,** $\bar{\eta}$ **^a^** | **Origin of Env** |
| --- | --- | --- | --- | --- | --- |
| JR-FL | wt | 2 | 2 | 11.8 | Ref [12] |
| JR-FL | ΔV1V2 | 3 | 4 | 6.9 | Ref [13] |
| JR-FL | D664N | 2 | 2 | 11.8 | This study |
| JR-FL | V549M N554D | 4 | 6 | 11.8 | This study |
| JR-FL | N332S P369L M373R D664N | 2 | 2 | 11.8 | This study |
| JR-FL | ΔCT | N.D. | N.D. | 19.4 | Ref [14] |
| JR-CSF | wt | 2 | 2 | 11.8 | Ref [12] |
| NL4-3 | wt | 7 | 4 | 13.5 | Ref [15] |
| NL4-3 | ΔV1V2 | 8 | 6 | 11.9 | Ref [13] |
| ZA110 | wt | 2 | 2 | 15 | Ref [13] |
| ZA110 | ΔV1V2 | 9 | N.D. | 11.9 | Ref [13] |
| SF162 | wt | 5 | 4 | 16 | Ref [16] |
| BG505 | wt | 3 | 2 | 9.5 | Ref [17] |
| P3N | wt | 2 | 2 | 20.3 | Ref [18] |

^a^ T and $\bar{\eta}$ were estimated in Brandenberg *et al.,* 2015 [1].

N.D., not determined
