# Supplementary material for: Predicting HIV-1 transmission and antibody neutralization efficacy in vivo from stoichiometric parameters
Source: PLoS Pathog. 2017 May 4;13(5):e1006313. doi: 10.1371/journal.ppat.1006313 (PMC5417720; doi:10.1371/journal.ppat.1006313)
Supplement: S4 Table — (DOCX) [file ppat.1006313.s020.docx]

**S4 Table: Antibodies employed in this study**

| **Antibody** | **Epitope** | **Origin** |
| --- | --- | --- |
| b12 | CD4 binding site | Ref [19] |
| b6 | CD4 binding site | Ref [19] |
| PGV04 | CD4 binding site | Ref [20] |
| VRC01 | CD4 binding site | Ref [21] |
| NIH45.46 | CD4 binding site | Ref [22] |
| PG9 | Quaternary: V1V2 loop - glycan | Ref [23] |
| PGT121 | Glycan | Ref [24] |
| PGT128 | Glycan | Ref [24] |
| PGT135 | Glycan | Ref [24] |
| PGT145 | Quaternary: V1V2 loop - glycan | Ref [24] |
| 17b | CD4-induced | Ref [25] |
| 48D | CD4-induced | Ref [25] |
| 447-52D | V3 loop | Ref [26] |
| 1-79 | V3 loop | Ref [27] |
| 2G12 | Glycan | Ref [28] |
| 2F5 | Membrane proximal external region (MPER) | Ref [29] |
