# Supplementary material for: Predicting HIV-1 transmission and antibody neutralization efficacy in vivo from stoichiometric parameters
Source: PLoS Pathog. 2017 May 4;13(5):e1006313. doi: 10.1371/journal.ppat.1006313 (PMC5417720; doi:10.1371/journal.ppat.1006313)
Supplement: S5 Table — (DOCX) [file ppat.1006313.s021.docx]

**S5 Table: Antibody IC50 and K_D_**

| **Antibody** | **IC50^a^**  Sanders *et a*l., 2013 [30], µg/ml | **IC50^a^**  Group Trkola, µg/ml | **IC50^b^**  Hoffenberg *et a*l., 2013 [31], µg/ml | **Mean IC50** µg/ml | **K_D_^a^**  Yasmeen *et al.,* 2014 [4]_,_ nM |
| --- | --- | --- | --- | --- | --- |
| VRC01 | 0.07 | 0.085 | 0.06 | 0.078 | 0.72 |
| PGV04 | 0.066 | 0.103 | 0.05 | 0.085 | 0.26^c^ |
| PGT121 | 0.015 | 0.049 | 0.06 | 0.032 | 0.76 |
| PGT123 | 0.028 | N.D. | 5.37 | 0.028 | 1.11 |
| PGT145 | 0.083 | 0.071 | < 0.01 | 0.077 | 2.9 |
| 2G12 | 0.79 | 0.426 | >25 | 0.608 | 8.65^d^ |

^a^ Both Sanders *et al.,* 2013 [30] and Yasmeen *et al*., 2014 [4] employed BG505 T332N in their studies, reconstituting an N-linked glycosylation site important for glycan-targeting antibodies. This Env was also used in this study to determine IC50´s.

^b^ Hoffenberg *et al.,* 2013 [31] employed BG505 without the T332N mutation. Since this glycosylation site influences binding and neutralization of glycan-targeting antibodies (notably, 2G12, and the PGT nAbs), we provide the IC50´s here for comparison only (grey shading).

^c^ Extrapolated from Table S2 in Yasmeen *et al.,* 2014 [4].

^d^ Listed as the average for 2G12 K_D_’s reported by Yasmeen *et al*., 2014 [4] and Sanders *et al*., 2013 [30] due to potential confounding effects of using 2G12-purified BG505 trimers in the analysis of K_D._
