# Supplementary material for: Predicting HIV-1 transmission and antibody neutralization efficacy in vivo from stoichiometric parameters
Source: PLoS Pathog. 2017 May 4;13(5):e1006313. doi: 10.1371/journal.ppat.1006313 (PMC5417720; doi:10.1371/journal.ppat.1006313)
Supplement: S6 Table — (DOCX) [file ppat.1006313.s022.docx]

**S6 Table: Parameters of macaque passive antibody immunization vaginal challenge studies**

| **b12 study** [9] |  |
| --- | --- |
| b12 immunization regime | Weekly i.v. doses at 1 mg/kg |
| Average b12 serum concentration at challenge | 41.8 µg/ml |
| Average b12 mucosal concentration at challenge | 0.47 µg/ml (extrapolated, see S7 Table) |
| b12 IC50 for SHIV-P3 challenge virus: | 0.36 µg/ml [10]  2 µg/ml [8]  1.6 µg/ml [11]  Mean: 1.32 µg/ml |
| b12 extrapolated K_D_ for P3 | 7.08x10^-09^ M |
| Challenge regime | SHIV-P3  11 bi-weekly challenges with 3 TCID50, 397 500 virions  40 bi-weekly challenges with 10 TCID50, 1 325 000 virions |
| Infections | 1 animal infected after 6 3 TCID50 challenges  3 animals infected after 6, 23, 38 10 TCID50 challenges  1 animal uninfected after all challenges |
|  |  |
| **2G12 study** [8] |  |
| 2G12 immunization regime | Single dose 1 day pre-challenge at 40 mg/kg |
| Average 2G12 serum concentration at challenge | 1053 µg/ml |
| Average 2G12 mucosal concentration at challenge | 12 µg/ml (extrapolated, see S7 Table) |
| 2G12 IC50 for SHIV-P3 challenge virus: | 20 µg/ml [8] |
| 2G12 extrapolated K_D_ for P3 | 1.07x10^-07^ M |
| Challenge regime | SHIV-P3  Single dose, 500 TCID50, 66 250 000 virions |
| Infections | 3 animals protected, 2 infected |
|  |  |
| **PGT121 study** [10] |  |
| PGT121 immunization regime | Single dose 1 day pre-challenge at 5 / 1 / 0.2 mg/kg |
| Average PGT121 serum concentrations at challenge | 95 µg/ml for 5 mg/kg immunization  15 µg/ml for 1 mg/kg immunization  1.8 µg/ml for 0.2 mg/kg immunization |
| Average PGT121 mucosal concentrations at challenge | 0.9 µg/ml for 5 mg/kg immunization  0.2 µg/ml for 1 mg/kg immunization  0.02 µg/ml for 0.2 mg/kg immunization (extrapolated, see S7 Table) |
| PGT121 IC50 for SHIV-P3 challenge virus: | 0.002 µg/ml [10]  0.03 µg/ml [11]  Mean : 0.016 µg/ml |
| PGT121 extrapolated K_D_ for P3 | 8.59x10^-11^ M |
| Challenge regime | SHIV-P3  Single dose, 300 TCID50, 39 750 000 virions |
| Infections | 5 of 5 animals protected for 5 and 1 mg/kg immunizations  3 animals protected, 2 infected for 0.2 mg/kg immunization |

| **PGT126 study** [11] |  |
| --- | --- |
| PGT126 immunization regime | Single dose 1 day pre-challenge at 10 / 2 / 0.4 mg/kg |
| Average PGT126 serum concentrations at challenge | 98 µg/ml for 10 mg/kg immunization  20 µg/ml for 2 mg/kg immunization  3.6 µg/ml for 0.4 mg/kg immunization |
| Average PGT126 mucosal concentrations at challenge | 1.1 µg/ml for 10 mg/kg immunization  0.22 µg/ml for 2 mg/kg immunization  0.04 µg/ml for 0.4 mg/kg immunization (all data extrapolated, see S7 Table) |
| PGT126 IC50 for SHIV-P3 challenge virus: | 0.3 µg/ml [11] |
| PGT126 extrapolated K_D_ for P3 | 1.61x10^-09^ M |
| Challenge regime | SHIV-P3  Single dose, 300 TCID50, 39 750 000 virions |
| Infections | 5 of 5 animals protected for 10 mg/kg immunizations,  2 animals protected, 3 infected for 2 mg/kg immunization, 1 animal protected, 4 infected for 0.4 mg/kg immunization |
