# Supplementary material for: Predicting HIV-1 transmission and antibody neutralization efficacy in vivo from stoichiometric parameters
Source: PLoS Pathog. 2017 May 4;13(5):e1006313. doi: 10.1371/journal.ppat.1006313 (PMC5417720; doi:10.1371/journal.ppat.1006313)
Supplement: S7 Table — (DOCX) [file ppat.1006313.s023.docx]

**S7 Table: Extrapolation of vaginal antibody concentrations in macaque challenge studies**

| **b12 study** [9] | |  |  |
| --- | --- | --- | --- |
| **b12 immunizations** | **b12 serum concentration** | **b12 vaginal concentration** | **Factor serum : vaginal Ab concentration^a^** |
| 1 mg/kg | 41.8 µg/ml | N.D.  0.47 µg/ml extrapolated | The 0.47 µg/ml vaginal b12 concentration were extrapolated from the serum concentration assuming a factor of 89 (the mean of the factors of 90.3 and 87.8 obtained in the PGT121 and 2G12 studies below) for serum-to-vaginal nAb concentration. |

| **2G12 study** [8] | |  |  |
| --- | --- | --- | --- |
| **2G12 immunizations** | **2G12 serum concentration** | **2G12 vaginal concentration** | **Factor serum : vaginal Ab concentration^a^** |
| 40 mg/kg | 1053 µg/ml | N.D.  12 µg/ml extrapolated | 87.8  The 12 µg/ml vaginal concentration were extrapolated from the 5 mg/kg test immunization, assuming a 8-fold higher dose (40 mg/kg compared to 5 mg/kg) and a linear relationship between immunization dose and serum-to-vaginal nAb concentration. |
| 5 mg/kg test immunization  (no challenge) | N.D. | 1.5 µg/ml | N.D. |

| **PGT121 study** [10] | |  |  |
| --- | --- | --- | --- |
| **PGT121 immunizations** | **PGT121 serum concentration** | **PGT121 vaginal concentration** | **Factor serum : vaginal Ab concentration^a^** |
| 5 mg/kg | 95 µg/ml | 0.9 µg/ml | 105.6 |
| 1 mg/kg | 15 µg/ml | 0.2 µg/ml | 75 |
| 0.2 mg/kg | 1.8 µg/ml | N.D. (below study detection limit).  0.02 µg/ml extrapolated | Assuming an average factor of 90.3 for serum-to-vaginal nAb concentration derived from the 5 and 1 mg/kg immunizations (factors 105.6 and 75), we obtain 0.02 µg/ml vaginal PGT121 concentration for the 0.2 mg/kg immunization. |

| **PGT126 study** [11] | |  |  |
| --- | --- | --- | --- |
| **PGT126immunizations** | **PGT126 serum concentration** | **PGT126 vaginal concentration** | **Factor serum : vaginal Ab concentration^a^** |
| 10 mg/kg | 98 µg/ml | N.D.  1.1 µg/ml extrapolated | 89 (the mean of the factors of 90.3 and 87.8 obtained in the PGT121 and 2G12 studies above) for serum-to-vaginal nAb concentration. |
| 2 mg/kg | 20 µg/ml | N.D.  0.22 µg/ml extrapolated | 89 |
| 0.4 mg/kg | 3.6 µg/ml | N.D.  0.04 µg/ml extrapolated | 89 |

**^a^** The factors for serum-to-vaginal nAb concentrations listed for the above studies, in the range of 90:1, are in good agreement with previously published data [32-34].
