# Supplementary material for: Predicting HIV-1 transmission and antibody neutralization efficacy in vivo from stoichiometric parameters
Source: PLoS Pathog. 2017 May 4;13(5):e1006313. doi: 10.1371/journal.ppat.1006313 (PMC5417720; doi:10.1371/journal.ppat.1006313)
Supplement: S1 References — (DOCX) [file ppat.1006313.s024.docx]

**S1 References**

1. Brandenberg OF, Magnus C, Rusert P, Regoes RR, Trkola A. Different Infectivity of HIV-1 Strains Is Linked to Number of Envelope Trimers Required for Entry. PLoS Pathog. 2015;11(1):e1004595. Epub 2015/01/09. doi: 10.1371/journal.ppat.1004595. PubMed PMID: 25569556; PubMed Central PMCID: PMCPmc4287578.

2. Magnus C, Rusert P, Bonhoeffer S, Trkola A, Regoes RR. Estimating the stoichiometry of human immunodeficiency virus entry. J Virol. 2009;83(3):1523-31. Epub 2008/11/21. doi: 10.1128/jvi.01764-08. PubMed PMID: 19019953; PubMed Central PMCID: PMC2620894.

3. Zhu P, Liu J, Bess J, Jr., Chertova E, Lifson JD, Grise H, et al. Distribution and three-dimensional structure of AIDS virus envelope spikes. Nature. 2006;441(7095):847-52. Epub 2006/05/27. doi: 10.1038/nature04817. PubMed PMID: 16728975.

4. Yasmeen A, Ringe R, Derking R, Cupo A, Julien JP, Burton DR, et al. Differential binding of neutralizing and non-neutralizing antibodies to native-like soluble HIV-1 Env trimers, uncleaved Env proteins, and monomeric subunits. Retrovirology. 2014;11:41. doi: 10.1186/1742-4690-11-41. PubMed PMID: 24884783.

5. Pilcher CD, Joaki G, Hoffman IF, Martinson FE, Mapanje C, Stewart PW, et al. Amplified transmission of HIV-1: comparison of HIV-1 concentrations in semen and blood during acute and chronic infection. AIDS. 2007;21(13):1723-30. doi: 10.1097/QAD.0b013e3281532c82. PubMed PMID: 17690570.

6. Rehan N, Sobrero AJ, Fertig JW. The semen of fertile men: statistical analysis of 1300 men. Fertil Steril. 1975;26(6):492-502. Epub 1975/06/01. PubMed PMID: 1169171.

7. Carias AM, McCoombe S, McRaven M, Anderson M, Galloway N, Vandergrift N, et al. Defining the interaction of HIV-1 with the mucosal barriers of the female reproductive tract. J Virol. 2013;87(21):11388-400. Epub 2013/08/24. doi: 10.1128/jvi.01377-13. PubMed PMID: 23966398; PubMed Central PMCID: PMCPmc3807311.

8. Hessell AJ, Rakasz EG, Poignard P, Hangartner L, Landucci G, Forthal DN, et al. Broadly neutralizing human anti-HIV antibody 2G12 is effective in protection against mucosal SHIV challenge even at low serum neutralizing titers. PLoS Pathog. 2009;5(5):e1000433. Epub 2009/05/14. doi: 10.1371/journal.ppat.1000433. PubMed PMID: 19436712; PubMed Central PMCID: PMCPmc2674935.

9. Hessell AJ, Poignard P, Hunter M, Hangartner L, Tehrani DM, Bleeker WK, et al. Effective, low-titer antibody protection against low-dose repeated mucosal SHIV challenge in macaques. Nat Med. 2009;15(8):951-4. Epub 2009/06/16. doi: 10.1038/nm.1974. PubMed PMID: 19525965.

10. Moldt B, Rakasz EG, Schultz N, Chan-Hui PY, Swiderek K, Weisgrau KL, et al. Highly potent HIV-specific antibody neutralization in vitro translates into effective protection against mucosal SHIV challenge in vivo. Proc Natl Acad Sci U S A. 2012;109(46):18921-5. Epub 2012/10/27. doi: 10.1073/pnas.1214785109. PubMed PMID: 23100539; PubMed Central PMCID: PMCPmc3503218.

11. Moldt B, Le KM, Carnathan DG, Whitney JB, Schultz N, Lewis MG, et al. Neutralizing antibody affords comparable protection against vaginal and rectal simian/human immunodeficiency virus challenge in macaques. Aids. 2016;30(10):1543-51. Epub 2016/06/01. doi: 10.1097/qad.0000000000001102. PubMed PMID: 27243773; PubMed Central PMCID: PMCPMC4915739.

12. Koyanagi Y, Miles S, Mitsuyasu RT, Merrill JE, Vinters HV, Chen IS. Dual infection of the central nervous system by AIDS viruses with distinct cellular tropisms. Science. 1987;236(4803):819-22. Epub 1987/05/15. PubMed PMID: 3646751.

13. Rusert P, Krarup A, Magnus C, Brandenberg OF, Weber J, Ehlert AK, et al. Interaction of the gp120 V1V2 loop with a neighboring gp120 unit shields the HIV envelope trimer against cross-neutralizing antibodies. J Exp Med. 2011;208(7):1419-33. Epub 2011/06/08. doi: 10.1084/jem.20110196. PubMed PMID: 21646396; PubMed Central PMCID: PMCPmc3135368.

14. Brandenberg OF, Rusert P, Magnus C, Weber J, Boni J, Gunthard HF, et al. Partial rescue of V1V2 mutant infectivity by HIV-1 cell-cell transmission supports the domain`s exceptional capacity for sequence variation. Retrovirology. 2014;11(1):75. Epub 2014/10/08. doi: 10.1186/preaccept-1148975491133162. PubMed PMID: 25287422.

15. Adachi A, Gendelman HE, Koenig S, Folks T, Willey R, Rabson A, et al. Production of acquired immunodeficiency syndrome-associated retrovirus in human and nonhuman cells transfected with an infectious molecular clone. J Virol. 1986;59(2):284-91. Epub 1986/08/01. PubMed PMID: 3016298; PubMed Central PMCID: PMCPmc253077.

16. Cheng-Mayer C, Levy JA. Distinct biological and serological properties of human immunodeficiency viruses from the brain. Ann Neurol. 1988;23 Suppl:S58-61. Epub 1988/01/01. PubMed PMID: 3258140.

17. Wu X, Parast AB, Richardson BA, Nduati R, John-Stewart G, Mbori-Ngacha D, et al. Neutralization escape variants of human immunodeficiency virus type 1 are transmitted from mother to infant. J Virol. 2006;80(2):835-44. Epub 2005/12/28. doi: 10.1128/jvi.80.2.835-844.2006. PubMed PMID: 16378985; PubMed Central PMCID: PMCPMC1346878.

18. Ho SH, Tasca S, Shek L, Li A, Gettie A, Blanchard J, et al. Coreceptor switch in R5-tropic simian/human immunodeficiency virus-infected macaques. J Virol. 2007;81(16):8621-33. Epub 2007/06/01. doi: 10.1128/jvi.00759-07. PubMed PMID: 17537860; PubMed Central PMCID: PMC1951359.

19. Burton DR, Barbas CF, 3rd, Persson MA, Koenig S, Chanock RM, Lerner RA. A large array of human monoclonal antibodies to type 1 human immunodeficiency virus from combinatorial libraries of asymptomatic seropositive individuals. Proc Natl Acad Sci U S A. 1991;88(22):10134-7. Epub 1991/11/15. PubMed PMID: 1719545; PubMed Central PMCID: PMC52882.

20. Falkowska E, Ramos A, Feng Y, Zhou T, Moquin S, Walker LM, et al. PGV04, an HIV-1 gp120 CD4 binding site antibody, is broad and potent in neutralization but does not induce conformational changes characteristic of CD4. J Virol. 2012;86(8):4394-403. Epub 2012/02/22. doi: 10.1128/jvi.06973-11. PubMed PMID: 22345481; PubMed Central PMCID: PMCPmc3318667.

21. Wu X, Yang ZY, Li Y, Hogerkorp CM, Schief WR, Seaman MS, et al. Rational design of envelope identifies broadly neutralizing human monoclonal antibodies to HIV-1. Science. 2010;329(5993):856-61. Epub 2010/07/10. doi: science.1187659 [pii] 10.1126/science.1187659. PubMed PMID: 20616233.

22. Scheid JF, Mouquet H, Ueberheide B, Diskin R, Klein F, Olivera TY, et al. Sequence and Structural Convergence of Broad and Potent HIV Antibodies That Mimic CD4 Binding. Science. 2011. doi: 10.1126/science.1207227. PubMed PMID: 21764753.

23. Walker LM, Phogat SK, Chan-Hui PY, Wagner D, Phung P, Goss JL, et al. Broad and potent neutralizing antibodies from an African donor reveal a new HIV-1 vaccine target. Science. 2009;326(5950):285-9. Epub 2009/09/05. doi: 1178746 [pii] 10.1126/science.1178746. PubMed PMID: 19729618.

24. Walker LM, Huber M, Doores KJ, Falkowska E, Pejchal R, Julien JP, et al. Broad neutralization coverage of HIV by multiple highly potent antibodies. Nature. 2011;477(7365):466-70. Epub 2011/08/19. doi: 10.1038/nature10373. PubMed PMID: 21849977; PubMed Central PMCID: PMCPmc3393110.

25. Thali M, Moore JP, Furman C, Charles M, Ho DD, Robinson J, et al. Characterization of conserved human immunodeficiency virus type 1 gp120 neutralization epitopes exposed upon gp120-CD4 binding. J Virol. 1993;67(7):3978-88. Epub 1993/07/01. PubMed PMID: 7685405; PubMed Central PMCID: PMC237765.

26. Zolla-Pazner S, Zhong P, Revesz K, Volsky B, Williams C, Nyambi P, et al. The cross-clade neutralizing activity of a human monoclonal antibody is determined by the GPGR V3 motif of HIV type 1. AIDS Res Hum Retroviruses. 2004;20(11):1254-8. Epub 2004/12/14. doi: 10.1089/0889222042545054. PubMed PMID: 15588347.

27. Scheid JF, Mouquet H, Feldhahn N, Seaman MS, Velinzon K, Pietzsch J, et al. Broad diversity of neutralizing antibodies isolated from memory B cells in HIV-infected individuals. Nature. 2009;458(7238):636-40. Epub 2009/03/17. doi: nature07930 [pii] 10.1038/nature07930. PubMed PMID: 19287373.

28. Trkola A, Purtscher M, Muster T, Ballaun C, Buchacher A, Sullivan N, et al. Human monoclonal antibody 2G12 defines a distinctive neutralization epitope on the gp120 glycoprotein of human immunodeficiency virus type 1. J Virol. 1996;70(2):1100-8. Epub 1996/02/01. PubMed PMID: 8551569; PubMed Central PMCID: PMCPmc189917.

29. Muster T, Steindl F, Purtscher M, Trkola A, Klima A, Himmler G, et al. A conserved neutralizing epitope on gp41 of human immunodeficiency virus type 1. J Virol. 1993;67(11):6642-7. Epub 1993/11/01. PubMed PMID: 7692082; PubMed Central PMCID: PMCPmc238102.

30. Sanders RW, Derking R, Cupo A, Julien JP, Yasmeen A, de Val N, et al. A next-generation cleaved, soluble HIV-1 Env Trimer, BG505 SOSIP.664 gp140, expresses multiple epitopes for broadly neutralizing but not non-neutralizing antibodies. PLoS Pathog. 2013;9(9):e1003618. Epub 2013/09/27. doi: 10.1371/journal.ppat.1003618. PubMed PMID: 24068931; PubMed Central PMCID: PMCPmc3777863.

31. Hoffenberg S, Powell R, Carpov A, Wagner D, Wilson A, Kosakovsky Pond S, et al. Identification of an HIV-1 clade A envelope that exhibits broad antigenicity and neutralization sensitivity and elicits antibodies targeting three distinct epitopes. J Virol. 2013;87(10):5372-83. doi: 10.1128/jvi.02827-12. PubMed PMID: 23468492.

32. Li Q, Zeng M, Duan L, Voss JE, Smith AJ, Pambuccian S, et al. Live simian immunodeficiency virus vaccine correlate of protection: local antibody production and concentration on the path of virus entry. J Immunol. 2014;193(6):3113-25. doi: 10.4049/jimmunol.1400820. PubMed PMID: 25135832.

33. Shingai M, Donau OK, Plishka RJ, Buckler-White A, Mascola JR, Nabel GJ, et al. Passive transfer of modest titers of potent and broadly neutralizing anti-HIV monoclonal antibodies block SHIV infection in macaques. J Exp Med. 2014;211(10):2061-74. Epub 2014/08/27. doi: 10.1084/jem.20132494. PubMed PMID: 25155019; PubMed Central PMCID: PMCPmc4172223.

34. Klein K, Veazey RS, Warrier R, Hraber P, Doyle-Meyers LA, Buffa V, et al. Neutralizing IgG at the portal of infection mediates protection against vaginal simian/human immunodeficiency virus challenge. J Virol. 2013;87(21):11604-16. doi: 10.1128/jvi.01361-13. PubMed PMID: 23966410.
